# Propofol addiction drives neuronal senescence and cognitive decline via autophagy-mediated ADAR1/SIRT1 disruption

**DOI:** 10.1038/s42003-025-09388-8

**Published:** 2025-12-22

**Authors:** Xidi Wang, Shounuo Lin, Zhiting Zou, Dan Zhu, Jiahui Zhou, Xiaoyu Zhang, Jianan Lv, Wenhua Zhou, Yu Liu, Zizhen Si

**Affiliations:** 1 Medical Research Center, The First Affiliated Hospital of Ningbo University, Ningbo, PR China; 2 School of Basic Medical Sciences, Health Science Center, Ningbo University, Ningbo, China; 3 Addiction treatment laboratory of Zhejiang, Hangzhou, China; 4 Department of Psychology, Collage of Teacher Education, Ningbo University, Ningbo, China; 5 School of Pharmacy, Health Science Center, Ningbo University, Ningbo, China; 6 Department of Psychiatry, The Affiliated Kangning Hospital of Ningbo University, Ningbo, China

**Keywords:** Addiction, Neurotoxicity syndromes

## Abstract

Propofol addiction represents a significant clinical challenge with no approved pharmacotherapy. While cognitive decline is a hallmark of substance use disorders, its underlying mechanisms in propofol addiction remain unclear. This study investigates whether propofol abuse induces neuronal senescence and cognitive impairment and explores the involved molecular pathways. We found that propofol administration in mice led to significant learning and memory deficits, which were associated with p16^INK4a^-dependent neuronal senescence in the hippocampus. Knockdown of p16^INK4a^ alleviated both senescence and cognitive decline. Mechanistically, propofol triggered autophagic degradation of ADAR1 via LC3-binding motifs, leading to reduced SIRT1 expression and subsequent upregulation of p16^INK4a^. Both neuronal-specific and systemic inhibition of autophagy attenuated propofol-induced senescence, cognitive impairment, and addictive behaviors. Our findings reveal a novel ADAR1-SIRT1- p16^INK4a^ pathway mediated by autophagy in propofol addiction, suggesting that targeting autophagy or senescence may offer therapeutic strategies for treating propofol use disorder.

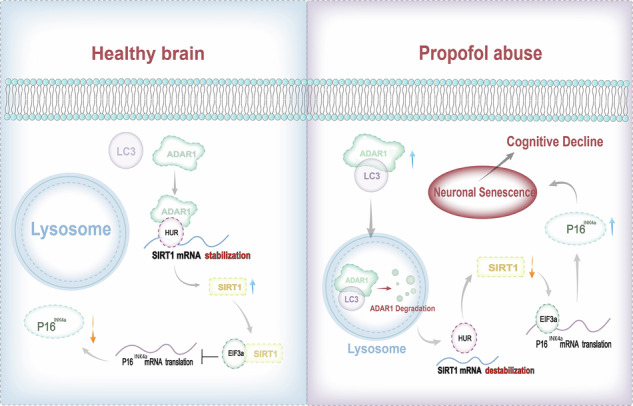

## Abstract (web summary)

Propofol addiction induces neuronal senescence and cognitive decline via an autophagy-mediated ADAR1-SIRT1-p16^INK4a^ pathway, suggesting autophagy/senescence as potential therapeutic targets.

## Introduction

Propofol, the cornerstone intravenous anesthetic enabling modern precision surgery^1^, has paradoxically emerged as a potent addictive substance with escalating abuse rates worldwide^2,3^. Clinical studies have reported that approximately 16.8% to 18.2%^4,5^ of patients undergoing propofol sedation develop irreversible cognitive deficits, suggesting its potential neurotoxicity. While its acute sedative effects are mediated primarily through γ-aminobutyric acid (GABA) receptors^6^, the molecular mechanisms underlying long-term cognitive impairment following repeated exposure remain poorly understood, hindering the development of effective interventions.

Cognitive impairment is a hallmark of addiction^7^, and cellular senescence, a state of irreversible cell cycle arrest, has recently been implicated in various mental disorder^8–10^. The cyclin-dependent kinase inhibitor p16^INK4a^ (encoded by CDKN2A), a key regulator of cellular senescence, has been identified as a highly specific biomarker of aging-related cognitive decline in both animal models and human postmortem studies^11,12^. However, whether anesthetic agents such as propofol induce neuronal senescence leading to cognitive deficits remains unexplored.

At the molecular level, adenosine deaminase acting on RNA-1 (ADAR1), an RNA-editing enzyme^13^, interacts with the deacetylase SIRT1 to help maintain chromatin stability and gene expression regulation. SIRT1 suppresses p16^INK4a^ expression through heterochromatin remodeling, while ADAR1 stabilizes SIRT1 mRNA via editing within its 3’ untranslated region^14^. Notably, both ADAR1 and SIRT1 can be degraded through autophagy under conditions of proteostatic stress^15,16^. Nevertheless, it remains unknown whether propofol addiction triggers autophagy-mediated degradation of the ADAR1/SIRT1 axis, thereby promoting neuronal senescence.

Based on this background, we hypothesize that propofol activates autophagy leading to ADAR1 degradation, disrupts SIRT1-mediated epigenetic repression, and induces p16^INK4a^-dependent neuronal senescence, ultimately resulting in cognitive impairment and addictive behaviors. To test this hypothesis, we employed behavioral assays, cellular models, and transgenic mice, and demonstrated that^1^: propofol upregulates p16^INK4a^ expression and induces senescence in hippocampal neurons, accompanied by cogni tive decline^2^; the ADAR1/SIRT1 pathway regulates p16^INK4a^ expression, and propofol enhances ADAR1 degradation via autophagy activation^3^; two LC3-interacting region (LIR) motifs within ADAR1 mediate its binding to the autophagy machinery; and ref. ^4^ either neuronal-specific or systemic inhibition of autophagy significantly ameliorates propofol-induced senescence, cognitive deficits, and addictive behaviors.

This study is the first to reveal the role of the autophagy–ADAR1–SIRT1–p16^INK4a^ axis in propofol-induced neuronal senescence and cognitive dysfunction. These findings not only provide novel insights into the neurotoxicity of anesthetics but also establish a theoretical foundation for developing senescence-targeting therapies against iatrogenic addiction.

## Materials and methods

### Animal models and ethical statement

The Cdkn2a^flox/flox^ (p16^INK4a flox/flox^) mice were (Cat. NO. NM-CKO-234392) were purchased from Shanghai Model Organisms Center, Inc. CaMKIIa-Cre mice were also provided by Shanghai model organisms. The Cdkn2a^flox/flox^ (p16^INK4a flox/flox^) mice were mated with the CaMKIIa-driven Cre recombinase transgenic mice, and a neuronal-specific p16^INK4a^-deficient mouse model (p16^INK4a^-CKO mice) was generated. Atg7^flox/flox^ mice (Cat. NO. NM-CKO-220047) were purchased from Shanghai Model Organisms Center, Inc. The ATG7^flox/flox^ mice were bred with the CaMKIIa-driven Cre recombinase transgenic mice, and a neuronal-specific ATG7-deficient mouse model (ATG7-CKO mice) was generated. All mice were bred in the Experimental Animal Central of Ningbo University, and mice were housed under a 12 h light/dark cycle with controlled humidity (40%–60%) and temperature (23 ± 1 °C). For autophagy inhibition, C57B/L6 mice received intranasal injection of Lys05 (10 mg/kg, Selleck, USA, Catalog S8369) once daily from the day of propofol exposure until the mice were sacrificed. All animals used in this study were 2-month-old male mice.

Wild-type C57BL/6 Mice:

These animals were used to establish the baseline phenotypical and molecular effects of chronic propofol exposure. This model is well-characterized in neuroscience research and provides a standardized genetic background, minimizing variability in behavioral and biochemical readouts related to cognitive function and cellular senescence.

p16^INK4a^ Knockdown Mice (p16^INK4a^-CKO mice):

To directly investigate the causal role of p16^INK4a^ in propofol-mediated senescence and cognitive decline, we employed a neuron-specific knockdown model. This allowed us to demonstrate that reduction of p16^INK4a^ expression rescues both synaptic and cognitive deficits, confirming its pivotal role in the pathway.

Autophagy-Deficient Mice (ATG7-CKO mice):

This neuron-specific autophagy knockout model was selected to interrogate the functional contribution of autophagy to ADAR1 degradation and subsequent senescence activation. Its use provides targeted genetic evidence that autophagic flux is necessary for propofol-induced senescence and behavioral impairment.

We have complied with all relevant ethical regulations for animal use. All animal experiments were performed in strict adherence to the National Institutes of Health Guidelines for the Care and Use of Laboratory Animals and received full ethical approval from the Institutional Animal Care and Use Committee at Ningbo University (Approval ID: NBU-IACUC-2021-10476).

### Animal sacrifice

For molecular and biochemical analyses: Mice were sacrificed by cervical dislocation under deep anesthesia (induced by isoflurane, R510-22-10, RWD life science, China) 24 hours after the last self-administration session to assess the sustained effects of propofol. For histological analyses: Mice were terminally anesthetized and underwent transcardial perfusion with ice-cold PBS followed by 4% paraformaldehyde (PFA, P0099-100ml, Beyotime Biotechnology, China) to fix the brains prior to extraction and sectioning.

### Surgery and propofol self-administration training

The study used propofol (Diprivan®, 10 mg/mL), which was provided by the Department of Anesthesiology at Ningbo University First Affiliated Hospital and dissolved in 0.9% saline. All animal procedures were carried out in full compliance with the Ningbo University-approved protocol (NBU-IACUC-2021-10476), Good Clinical Practice guidelines, and institutional policies for high-alert medications. Mice were anesthetized by intraperitoneal injection of 1.0% sodium pentobarbital (50 mg/kg). A silastic catheter (Dow Corning, 508-001) was surgically implanted into the jugular vein and connected subcutaneously to a cannula, which was attached to a polyethylene assembly mounted on the dorsal side of the mouse. To maintain catheter patency and prevent postoperative infection, the catheters were flushed daily with sterile saline containing 0.4% heparin sodium^17^. Mice were trained in operant chambers where a nose-poke in the left hole was designated as an active response, while the right hole served as an inactive control. Each active nose-poke under the fixed ratio 1 (FR1) schedule triggered an infusion of 2 mg/kg/infusion propofol, followed by a 20-second timeout period. Daily FR1 sessions lasted for 4 hours or until a maximum of 200 infusions was reached. Stable propofol SA was defined as achieving at least 25 infusions per session, an active-to-inactive response ratio greater than 2:1, and less than 20% variability in daily infusions over three consecutive sessions.

Following acquisition, mice underwent propofol SA under either FR1 or progressive ratio (PR) schedules. PR sessions extended for 6 hours daily, with the response requirement for each subsequent infusion increasing according to the formula: (Response ratio = [5e ^(injection number x0.18)^] – 5). The breakpoint under the PR schedule was defined as the highest number of nose-pokes completed for the final infusion before a 1-hour period without reinforcement. Mice proceeded to subsequent experiments only after the breakpoint stabilized, with less than 15% variability in infusions over three days.

### Morris water maze (MWM) test

A circular pool (diameter: 120 cm, height: 50 cm) was filled with milk-tinted water maintained at 25 ± 1 °C. A platform was hidden below the water surface in one pool quadrant. Mice were trained in four trials daily for five consecutive days, starting at different positions for each trial, and allowed to swim freely for 90 s. The time mice spent swimming to the platform was recorded as escape latency; however, if a mouse failed to find the platform within 90 s, the researcher directed and kept it on the platform for 15 s. Probe trials were performed after the last trial of the acquisition period, and the platform was removed. The mice were allowed to swim freely for 90 s. The time spent in the target quadrant that previously contained the platform, and the number of platform location crosses were recorded and the EthoVision XT software was used for tracking.

### Y-maze test

The Y-maze apparatus consisted of three identical opaque acrylic arms (each 40 cm long × 10 cm wide × 15 cm high) positioned at 120° angles. The test was conducted under dim light (∼50 lux) and low-noise conditions. Mice were individually placed at the end of one arm and allowed to freely explore the maze for 8 min. Behavior was recorded and analyzed using an automated video-tracking system (EthoVision XT). An arm entry was defined as the mouse placing all four paws into the arm. Spontaneous alternation was defined as consecutive entries into all three arms without repetition. The percentage alternation was calculated as: Alternation (%) = [(Number of spontaneous alternation)/ (Total number of arm entries - 2)] × 100%. Mice with fewer than 8 total arm entries were excluded from analysis due to insufficient exploratory activity.

### Novel object recognition (NOR)

The NOR test was performed in an open-field arena (50 × 50 × 40 cm) under consistent lighting (∼60 lux). During the training phase, two identical objects (e.g., glass cubes, 5 × 5 × 5 cm) were placed in two opposite corners, 10 cm from the walls. Each mouse was allowed to explore the objects freely for 10 min. After a 90-min retention interval, one object was replaced with a novel object of similar size but distinct shape/texture. The mouse was reintroduced for a 10-min test session. Exploration was defined as directing the nose toward the object within ≤2 cm; climbing or sitting on the object was not counted. Sessions were video-recorded and analyzed blind to treatment groups. The recognition index (RI) was calculated as: RI = Time spent exploring novel object/(Time spent exploring novel + familiar object) ×100%. A preference Index (PI) during the training phase was also assessed to confirm absence of innate bias: PI = Time exploring one identical object/Time exploring both identical objects× 100%. Objects and positions were counterbalanced across groups to avoid place preference.

### SA-β-gal staining

Cells were fixed with 3% paraformaldehyde in PBS (pH 6.0) and subsequently incubated in an X-gal staining solution containing 1 mg/ml 5-bromo-4-chloro-indolyl-β-D-galactopyranoside, 5 mM potassium ferrocyanide, 5 mM potassium ferricyanide, 150 mM NaCl, and 2 mM MgCl2 in PBS (pH 6.0) for 16–20 hours at 37 °C. After staining, images were captured using a dissecting microscope. The results shown are representative of three independent experiments, each demonstrating consistent staining patterns^18^.

### Immunofluorescence

Brain slices or cells were fixed with 4% paraformaldehyde for 20 minutes at room temperature and subsequently washed three times with phosphate-buffered saline (PBS). The samples were blocked with a solution of 10 mg/ml bovine serum albumin (BSA) in PBS for 1 hour. Primary antibody was applied to the brain slices and incubated at 4°C overnight. After incubation, the slices were washed three times with PBS, each wash lasting 10 minutes. The slices were then incubated with a fluorescent secondary antibody (Proteintech, Chicago, USA) in the dark for 2 hours. Following this, the slices were stained with 4′,6-diamidino-2-phenylindole (DAPI; Solarbio, Beijing, China) for 10 minutes to visualize nuclei. To preserve fluorescence, an anti-fade mounting medium was applied, and the slices were coverslipped. Imaging was conducted using a confocal laser scanning microscope (Leica TCS SP8, Germany)^19^. The primary antibodies used in this study included LC3 (14600-1-AP, 1:500), ADAR1 (14330-1-AP, 1:200), PML (21041-1-AP, 1:200), HIRA (83194-5-RR, 1:200) and macroH2A1.2 (61427, 1:50) from Proteintech.

### Cell culture

HT22 cells (Cellverse Co., Ltd, China) were cultured in DMEM (Hyclone) with 10% FBS (Gibco) and 100 units/mL penicillin/streptomycin. Hippocampi were dissected from newborn mice brains in ice-cold Hanks’ Balanced Salt Solution (HBSS, Gibco). The tissues were enzymatically digested with 0.25% trypsin-EDTA for 15 minutes at 37 °C, followed by termination of digestion using DMEM (Gibco) supplemented with 10% FBS (Gibco). The digested tissues were gently triturated with a fire-polished glass pipette to dissociate neurons. The cell suspension was centrifuged at 800 × *g* for 5 minutes, and resuspended in neuronal culture medium consisting of Neurobasal-A medium, 2% B-27 supplement, 0.5 mM glutamine, and 1% penicillin-streptomycin. Cells were plated onto poly-D-lysine-coated culture plates or coverslips at a density of 5 × 10⁴ cells/cm² and maintained in a humidified incubator at 37 °C with 5% CO₂. Half of the culture medium was replaced every 3 days. Neuronal cultures were typically used for experiments after 7–10 days in vitro^20^.

### Virus injection

We purchased pADV-U6-shCdkn2a-CMV-EGFP, pADV-U6-blank-CMV-EGFP, AAV-CMV-Cdkn2a-EGFP and AAV-blank-CMV-EGFP from Shanghai Genechem Co.,Ltd. The AAV vectors were stereotaxically injected into hippocampus with a glass pipette at a slow flow rate of 60 nl/min. After the viral injection, the pipette was withdrawn 10 min. The stereotaxic coordinates used for hippocampus injections are defined as follows: Anteroposterior (AP): −2.0 mm to −2.3 mm, Mediolateral (ML): ±1.4 mm to ±1.8 mm, Dorsoventral (DV): −1.4 mm to −1.8 mm below dura mater.

### Western blot and Immunoprecipitation

Protein samples (50 μg) were separated by SDS-Polyacrylamide Gel Electrophoresis (SDS-PAGE) and subsequently transferred onto a nitrocellulose membrane. The membrane was blocked with 5% non-fat milk in Tris-buffered saline containing 0.1% Tween-20 (TBST) for 1 hour at room temperature, followed by incubation with the appropriate primary antibody diluted in blocking buffer overnight at 4 °C. After washing three times with TBST, the membrane was incubated with horseradish peroxidase (HRP)-conjugated secondary antibody (anti-rabbit or anti-mouse) for 1 hour at room temperature. Immunoreactive bands were visualized using enhanced chemiluminescence (ECL) reagent (GE Healthcare, Little Chalfont, UK) and imaged with a ChemiDoc™ Imager (Bio-Rad). Image acquisition was performed under non-saturating exposure conditions, and no post-capture alterations were applied^18^.

Cells were lysed in ice-cold lysis buffer composed of 50 mM Tris (pH 8.0), 150 mM NaCl, 1% NP-40, 10 mM EDTA, 5% glycerol, 1 mM phenylmethylsulfonyl fluoride (PMSF), 10 µg/mL aprotinin, and 10 µg/mL leupeptin. For experiments performed under denaturing conditions, 1% SDS was included in the lysis buffer. Lysates were clarified by centrifugation at 16,000 × *g* for 25 minutes at 4 °C. To reduce non-specific binding, the clarified lysates were precleared by incubation with normal mouse or rabbit IgG bound to Protein A/G Sepharose beads for 2 hours at 4 °C with gentle rotation, followed by centrifugation at 18,000 × *g* for 10 minutes. One-tenth of each lysate was reserved as input control for subsequent Western blot analysis. Immunoprecipitation was performed by incubating the precleared lysates overnight at 4 °C with anti-Myc or anti-Flag antibody pre-conjugated to Protein A/G agarose beads. The immunoprecipitates were then collected and washed three times with ice-cold lysis buffer.

The primary antibodies used in this study included LC3 (14600-1-AP, 1:1000), ADAR1 (14330-1-AP, 1:1000), and β-actin (66009-1-Ig, 1:5000) from Proteintech. GPX4 (ab125066, 1:1000), SIRT1 (ab110304, 1:1000) were purchased from Abcam. LAMP-2a (51-2200, 1:1000) from Invitrogen. p16^INK4a^ (ZMS1072, 1:1000) was purchased from sigmaaldrich.

### Quantitative RT-PCR

Total RNA was isolated from cells or tissues using TRIzol reagent (Invitrogen, Carlsbad, CA, USA) through chloroform phase separation and isopropanol precipitation. RNA concentration and purity were determined spectrophotometrically. Complementary DNA (cDNA) was synthesized from 1 µg of total RNA using a High-Capacity cDNA Reverse Transcription Kit (Thermo Fisher Scientific, Waltham, MA, USA) in a 20 µL reaction volume under the following conditions: 25 °C for 10 minutes, 37 °C for 120 minutes, and 85 °C for 5 minutes.

Quantitative PCR was carried out using Power SYBR® Green PCR Master Mix (Thermo Fisher Scientific) on an Applied Biosystems 7500 Real-Time PCR System. Each 20 µL reaction contained 1 µL of cDNA template, 10 µL of Master Mix, 200 nM of each forward and reverse primer, and nuclease-free water. The amplification protocol consisted of an initial denaturation at 95 °C for 10 minutes, followed by 35 cycles of denaturation at 94°C for 20 seconds, annealing at 60°C for 20 seconds, and extension at 72°C for 35 seconds. Melting curve analysis was performed to confirm amplification specificity. Gene expression levels were normalized to GAPDH or β-actin as internal controls and analyzed using the 2^−ΔΔCT^ method.

Total RNA was extracted by TRIzol (Invitrogen, USA) and reverse transcribed using PrimeScript RT reagent Kit (Takara, Japan). qRT-PCR was performed on an ABI StepOne Plus using SYBR Green^®^ Premix Ex Taq (Takara, Japan) as described previously^18^. The relative gene expression level was normalized to GAPDH and then calculated by means of the 2^−ΔΔCT^ method. Primer sequences are listed in a new Supplementary Table 1.

### Approaches used to interrogate the ADAR1/SIRT1 pathway and autophagy

Validation of ADAR1 and SIRT1 Expression Changes:

Protein Level: Protein expression of ADAR1, SIRT1, and p16^INK4a^ was analyzed by Western blot. Hippocampal tissues or cultured neurons were lysed in RIPA buffer. Equal amounts of protein (20–30 μg) were separated by SDS-PAGE, transferred to PVDF membranes, and incubated overnight at 4 °C with primary antibodies: anti-ADAR1 (1:1000), anti-SIRT1 (1:1000), anti-p16^INK4a^ (1:1000), and anti-β-actin (1:5000) as a loading control. Band intensities were quantified using ImageJ software (NIH) and normalized to β-actin.

mRNA Level: Total RNA was extracted using TRIzol reagent. cDNA was synthesized with a Reverse Transcription Kit. mRNA levels were determined by qRT-PCR using SYBR Green Master Mix. The 2^−ΔΔCT^ method was used for relative quantification, with Gapdh as the reference gene. Primer sequences are listed in a new Supplementary Table 1.

Analysis of Autophagic Activity:

Autophagic flux was assessed by measuring the protein levels of LC3-II via Western blot (1:1000). The interaction between LC3 and ADAR1 was confirmed by Co-IP. Protein lysates were incubated with an anti-ADAR1 antibody or IgG control overnight at 4 °C, followed by protein A/G beads. The precipitated complexes were analyzed by Western blot using anti-LC3 and anti-ADAR1 antibodies.

Functional Validation through Gain- and Loss-of-Function Studies:

To establish a causal link, we employed both pharmacological and genetic approaches.

In vitro: Primary hippocampal neurons were transfected with ADAR1-overexpression plasmid or siRNA targeting Adar1 prior to propofol exposure. The efficiency of manipulation was confirmed by qRT-PCR and Western blot. In vivo: The functional role of autophagy was validated using neuronal-specific Atg7 knockout mice, which we have now described in greater detail above.

### Inclusion/exclusion criteria

All animals that underwent successful surgery and were confirmed to carry the intended genotype were included in the study. Animals were excluded from the analysis a priori if they met any of the following criteria^1^: post-surgical weight loss exceeded 20% of pre-surgical body weight^2^; exhibited signs of severe infection or poor health unrelated to the experimental manipulation^3^; catheter patency failure occurred prior to the completion of the behavioral experiment (verified by a rapid loss of righting reflex following a brief intravenous infusion of propofol); or^4^ technical failure during tissue processing or data acquisition. No data points were excluded during statistical analysis unless they were identified as significant outliers using the Grubbs’ test (alpha = 0.05), which was established as the sole statistical exclusion criterion a priori.

### Reporting exclusions

A total of 21 mice were excluded from the final dataset for the reasons stated above. Specifically, 8 mice were excluded due to catheter patency failure, 9 mice due to post-surgical complications, and 4 data points due to technical failures. The final n for each group reported in the results represents only those animals and data points that passed all inclusion criteria.

### Sample size

The sample size (n) for each experimental group is reported in the corresponding figure legend. All n values represent biological replicates, not technical replicates. Sample sizes were chosen based on our previous experience with these models and behavioral tests to ensure sufficient statistical power, and are consistent with those commonly employed in the field.

### Experimental design, randomisation, and blinding

Randomisation. Randomisation was used to allocate animals to treatment groupswithin each genotype. The randomisation sequence was generated using the =RAND() function in Microsoft Excel for each cohort of mice. For example, within a litter containing both p16^INK4a^-CKO and control littermates, each mouse was assigned a random number and then allocated to the Propofol or Saline treatment group based on this sequence, ensuring an even distribution of littermates across groups where possible.

Minimisation of Confounders. To minimise potential confounding factors, the following strategies were employed:

Cage Location: All cages within the animal facility were systematically rotated on different shelves of the rack every three days throughout the experiment to avoid location-specific effects.

Order of Treatments and Measurements: The order in which mice underwent behavioral testing and tissue collection was randomized daily using a lottery system. This was done to ensure that any time-of-day effects or experimenter fatigue were distributed evenly across all experimental groups.

Batch Effects: The entire experiment was conducted over multiple replicated cohorts. Animals from all genotypes and treatment groups were represented in each cohort to prevent the confounding of experimental results with batch-to-batch variability.

### Study protocol

A detailed study protocol outlining the primary research question, hypothesis, key experimental design, and planned analyses was prepared internally and approved by our institutional animal ethics committee prior to the commencement of the study (Approval ID: NBU-IACUC-2021-10476).

### Figure and graphical abstract creation

All schematic diagrams and graphical elements in Figs. 3L, 5M, and the Graphical Abstract were created from scratch by the authors using basic drawing software (Adobe Illustrator). No pre-existing templates, elements from databases, or third-party toolswere used. All content is original and owned by the authors.

### Statistics and reproducibility

All statistical analyses were performed using SPSS (version 20.0; IBM, USA). Data are presented as mean ± standard deviation (SD). Comparisons between two groups were made using an unpaired two-tailed Student’s t-test. For comparisons among three or more groups, one-way or two-way analysis of variance (ANOVA) was applied. A p-value of less than 0.05 was considered statistically significant. The specific test used in each experiment is indicated in the corresponding figure legend. All experiments were independently repeated at least three times with consistent results. The sample size (n) for each experiment represents biological replicates, defined as cells or animals derived from distinct cultures or individuals. No statistical methods were used to predetermine sample size; however, sample numbers were chosen based on common practices in the field and our previous experience to ensure adequate power. Data inclusion and exclusion criteria were pre-established; no outliers were excluded unless clearly resulting from technical error, and such instances were noted during analysis. Efforts were made to ensure reproducibility through rigorous experimental standardization, including the use of aliquoted reagents, controlled passage numbers for cell lines, and blinded assessment during data collection and analysis where feasible.

## Results

### Propofol addiction triggers cognitive decline and hippocampal neuronal senescence

**Fig. 1:**
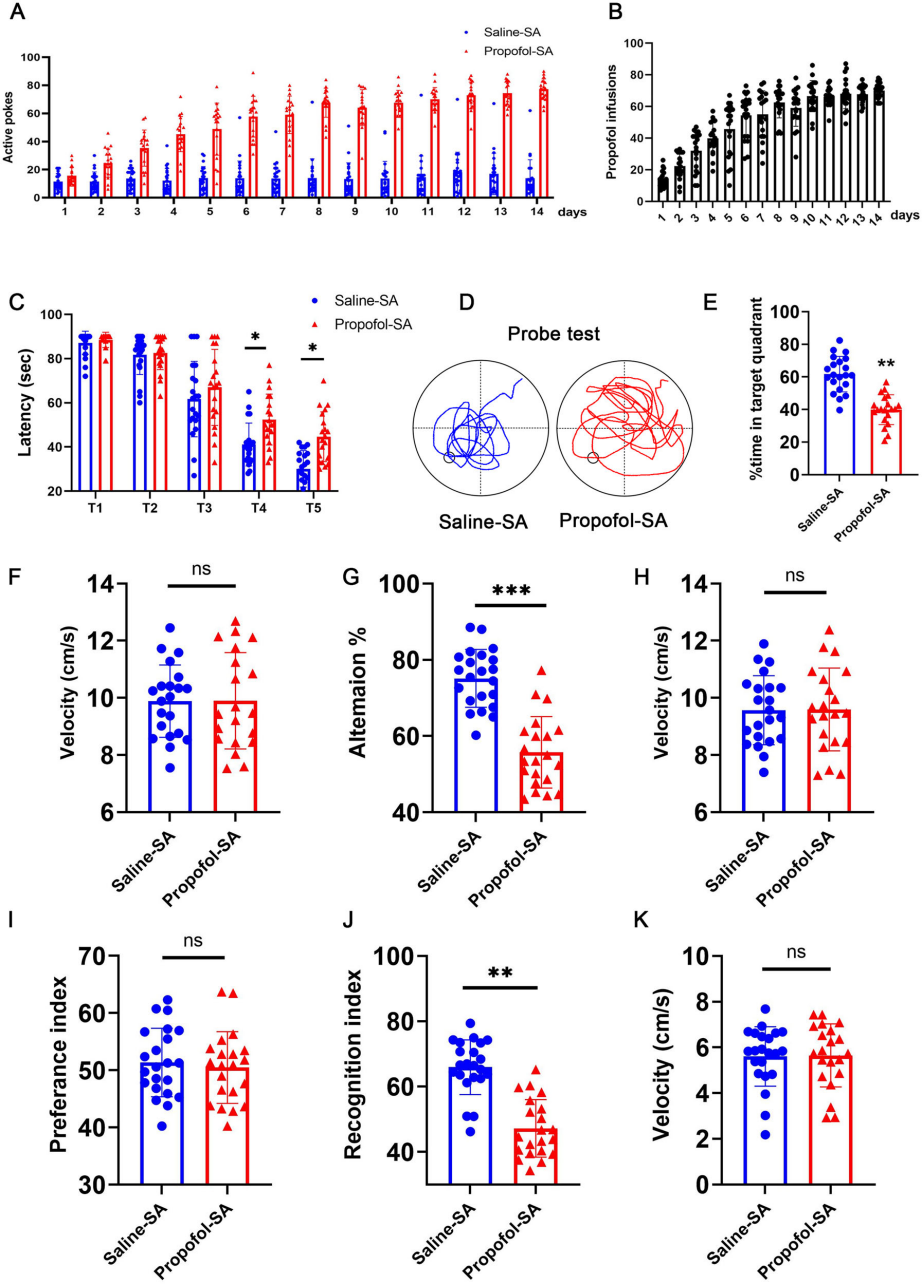
Propofol addiction induces cognitive decline. **A** Propofol-reinforced active nose-pokes and (**B**) Propofol-reinforced infusions under FR1 reinforcement. n = 21 mice/group. Condition A (Saline-SA): Mice self-administrated daily with saline. Condition B (Propofol-SA): Mice self-administrated daily with Propofol. **C** Escape latencies (s) of Saline-SA and Propofol-SA mice in the MWM test, and (**D**) Swim paths. **E** Percentage of time spent swimming in the target quadrant. **F** Average velocity during the probe test for mice in the MWM test. **G** Alternation of arms in the spontaneous alternation test of mice in the Y-maze test and (**H**) average velocity during the spontaneous alternation test of mice in the Y-maze test. Novel object recognition test (NOR). Preference index (**I**), recognition index (**J**), and (**K**) average velocity during the NOR test. For all figures: Values mean ± SD. Statistical significance was assessed using two-way ANOVA with post hoc Tukey’s multiple comparisons test. *P < 0.05, **P < 0.01, and ***P < 0.001.

To establish the causal link between propofol addiction and neurodegeneration, we employed a propofol self-administration paradigm (2 mg/kg/infusion, FR1 schedule) in adult C57BL/6 J mice over 14 days. Longitudinal behavioral tracking revealed escalating operant responding, with active nose poking numbers increasing from 17.3 ± 3.2 (day 1) to 72.3 ± 2.7 (day 14, p < 0.001 vs saline controls, Fig. 1A) and infusion numbers increasing from 16.6 ± 2.7 (day 1) to 64.1 ± 3.1 (day 14, Fig. 1B). Hippocampal-dependent memory deficits emerged on day 14, manifesting as impaired Morris water maze performance (propofol-SA group spent significantly more time in escape latency exploring the target quadrant than the saline-SA group, Fig. 1C-F), Y maze performance (the propofol-SA group exhibited a significantly lower rate of spontaneous alterations than those in the saline-SA group, Fig. 1G, H) and novel object recognition (the propofol-SA group showed a decreased RI than those in the saline-SA group, Fig. 1J, K).

**Fig. 2:**
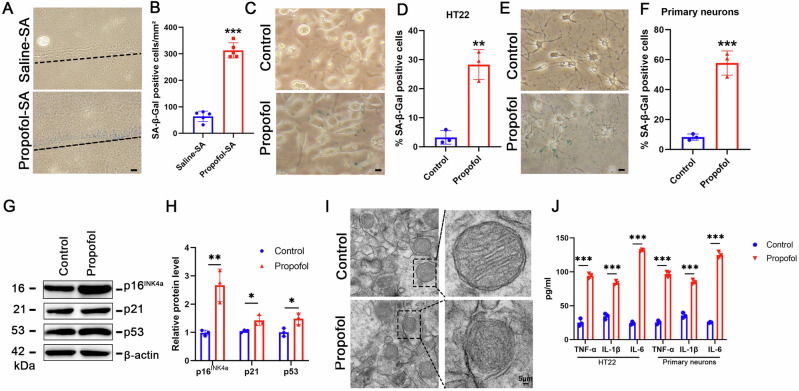
Propofol induces senescence in hippocampal neurons by upregulating p16^INK4a^ expression. **A** Representative SA-β-gal staining in hippocampus of saline-SA and propofol-SA mice. **B** Quantification of SA-β-gal positive cells. **C** Representative SA-β-gal staining in HT22 cells. **D** Quantification of SA-β-gal positive cells in the indicated HT22 cells. **E** Representative SA-β-gal staining in hippocampal-derived primary neurons. **F** Quantification of SA-β-gal positive cells in hippocampal-derived primary neurons. **G** Expression of p16^INK4a^, p21 and p53 were determined by Western blotting analysis in HT22 cells treated with propofol or saline (Control). **H** The relative expression of p16^INK4a^, p21 and p53 by normalizing against β-actin expression. **I** Typical neuronal images showed the mitochondria in the indicated HT22 cells. **J** TNF-α, IL-1β and IL-6 levels were detected in the supernatant of the indicated HT22 cells and hippocampal-derived primary neurons.

Multimodal analysis demonstrated profound senescence signatures in hippocampal subfields and hippocampal neurons. SA-β-gal+ cells increased 4.9-fold in CA1 (313.0 ± 28.9 vs 64.2 ± 19.8 cells/mm², p < 0.0001) compared to controls (Fig. 2A, B). Increased SA-β-gal activity was also observed in hippocampal neurons (both in hippocampal-derived HT-22 cell line and primary hippocampal neurons) after propofol treatment (Fig. 2C-F), with corresponding upregulation of p16^INK4a^ (2.7-fold, p < 0.01), p53 (1.48-fold, p < 0.05) and p21 (1.37-fold, p < 0.05) by immunoblotting (Fig. 2G, H). Transmission electron microscopy unveiled ultrastructural hallmarks of senescence of mitochondrial cristae dissolution (Fig. 2I). Senescence-associated secretory phenotype (SASP) emerged concomitantly, with hippocampal TNF-α, IL-1β, and IL-6 levels exceeding controls by 3.7-, 2.4-, and 5.5-fold respectively in HT22 cells and 3.8-, 2.4-, and 4.9-fold respectively in primary neurons (all p < 0.001) (Fig. 2J). These convergent behavioral and molecular-level findings establish propofol addiction as a novel driver of accelerated hippocampal senescence, potentially explaining long-term neurocognitive complications in clinical populations.

### Propofol upregulat×es p16^INK4a^ to drive senescence phenotype in hippocampal neurons

**Fig. 3:**
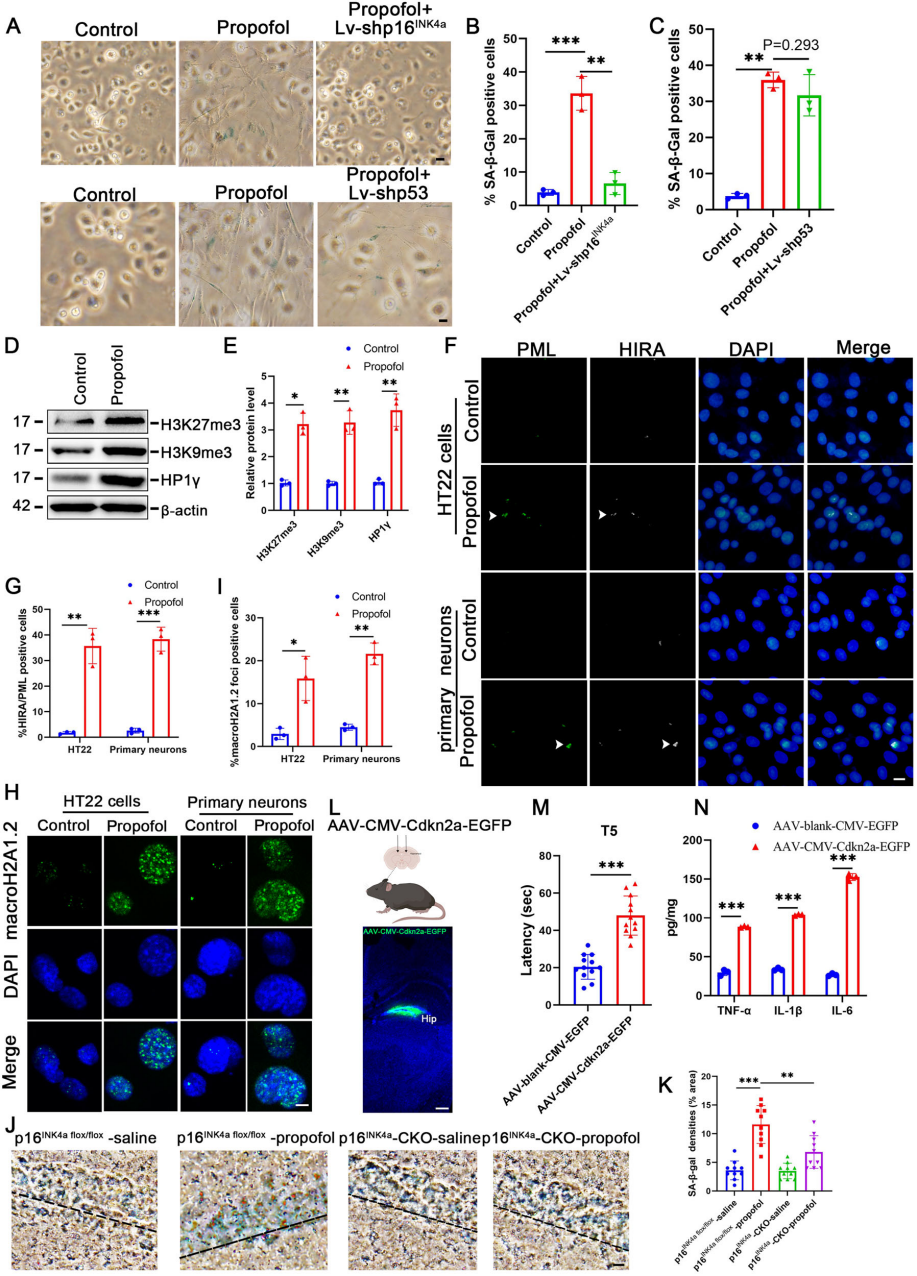
Propofol promotes p16^INK4a^ expression to induce hippocampal neuronal senescence. **A** Representative SA-β-gal staining in the indicated HT22 cells. **B**, **C** Quantification of SA-β-gal positive cells in the indicated HT22 cells. **D** Expression of H3K27me3, H3K9me3 and HP1γ were determined by Western blotting analysis in HT22 cells treated with propofol or saline (Control). **E** The relative expression of H3K27me3, H3K9me3 and HP1γ by normalizing against β-actin expression. Immunostaining HIRA and PML (**F**, **G**) and macroH2A1.2 **H**, **I** in the indicated HT22 cells and hippocampal-derived primary neurons. **J**, **K** Representative SA-β-gal staining and quantification of SA-β-gal positive cells in the hippocampus of indicated group. **L** A Schematic representation of viral injections into the hippocampus and representative IHC images of DAPI (blue) and GFP (green, viral marker) 6 weeks after viral injections. Scale bars represent 250 µm. **M** Escape latencies (s) of indicated groups in the MWM test. **N** TNF-α, IL-1β and IL-6 levels were detected in the hippocampus of indicated group. For all figures: Values mean ± SD. Statistical significance was assessed using two-way ANOVA with post hoc Tukey’s multiple comparisons test. *P < 0.05, **P < 0.01, and ***P < 0.001.

Mechanistic dissection revealed p16^INK4a^ as the central mediator of propofol-induced neuronal senescence. p16^INK4a^ p53 and p21 were all upregulated upon propofol treatment (Fig. 2G, H). However, only lentivirus-shRNA p16^INK4a^ (Lv-shp16^INK4a^) significantly reversed propofol induced the increase of SA-β-Gal activity (Fig. 3A–C). The upregulation of p16^INK4a^ in senescence is closely related to heterochromatin formation. We found the expression of H3K27me3, H3K9me3 and HP1γ, three heterochromatic markers, were upregulated in hippocampal-derived HT-22 cells after propofol treatment (Fig. 3D, E). HIRA and PML body pathway have been proved to mediate heterochromatin formation in senescence, and we found the increased colocalization of HIRA and PML bodies in propofol treatment cells, which correlated with the increased formation of SAHF as examined by macroH2A foci formation (Fig. 3F–I).

The Cdkn2a^flox/flox^ (p16^INK4a flox/flox^) mice were bred with the CaMKIIa-driven Cre recombinase transgenic mice, and a neuronal-specific p16^INK4a^-deficient mouse model (p16^INK4a^-CKO mice) was generated. The p16^INK4a^-CKO mice prevented senescence markers despite propofol exposure (Fig. 3J, K). Conversely, AAV-mediated p16^INK4a^ overexpression in naive mice recapitulated propofol-induced pathology, increasing SASP factors 3.0-3.1-5.8-fold (TNF-α: 88.57 ± 2.3 pg/mg, IL-1β: 104.0 ± 1.2 pg/mg, IL-6: 152.5 ± 2.7 pg/mg) and impairing spatial memory (water maze latency: 51.3 ± 4.1 s vs 23.8 ± 2.7 s, p < 0.001) (Fig. 3L–N). These data establish p16^INK4a^ upregulation as both necessary and sufficient for propofol-induced neurosenescence.

### Genetic ablation of p16^INK4a^ rescues propofol-induced neuronal senescence and cognitive impairment

**Fig. 4:**
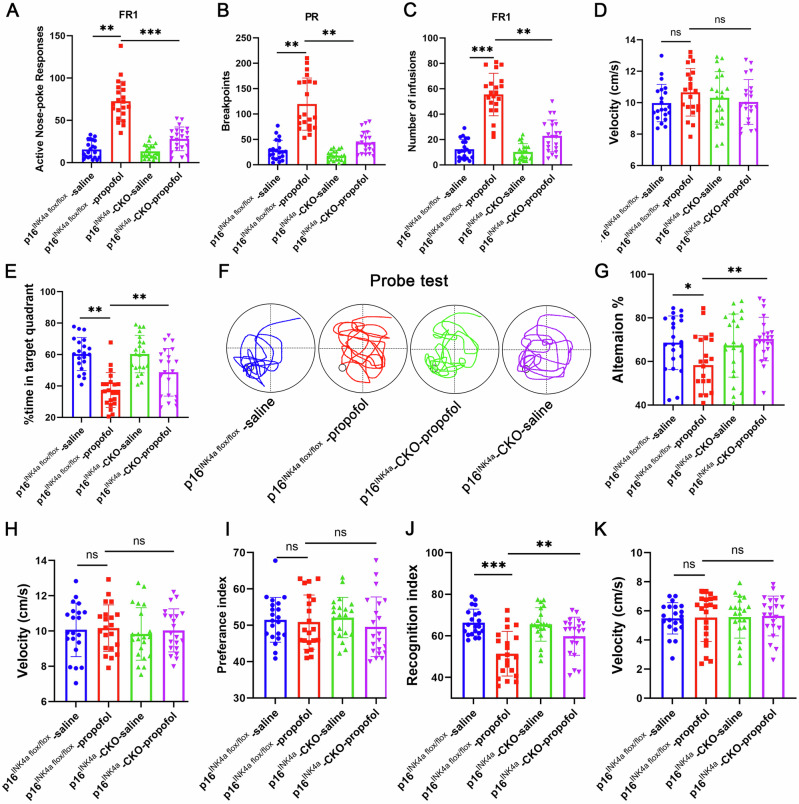
Inhibition of p16^INK4a^ prevents propofol-induced neuronal senescence and cognitive decline in p16^INK4a^-CKO mice. **A** Propofol-reinforced active nose-pokes and (**B**) Propofol-reinforced infusions under FR1 reinforcement. n = 21 mice/group. **C** The breakpoint number under PR reinforcement. n = 21 mice/group. **D** Average velocity during the probe test for mice in the MWM test. **E** Percentage of time spent swimming in the target quadrant, (**F**) Swim paths. **G** Alternation of arms in the spontaneous alternation test of mice in the Y-maze test and (**H**) average velocity during the spontaneous alternation test of mice in the Y-maze test. Novel object recognition test (NOR). Preference index (**I**), recognition index (**J**), and (**K**) average velocity during the NOR test. For all figures: Values mean ± SD. Statistical significance was assessed using two-way ANOVA with post hoc Tukey’s multiple comparisons test. *P < 0.05, **P < 0.01, and ***P < 0.001.

After 14 days of SA (Fig. 4A–C), the p16^INK4a^-CKO mice self-administered with propofol (p16^INK4a^-CKO-propofol) and p16^INK4a flox/flox^ mice self-administered with propofol (p16^INK4a flox/flox^ -propofol) were subjected to behavioral tests related to cognitive function. In the MWM test, the p16^INK4a^-CKO-propofol group spent less time exploring the target quadrant than the p16^INK4a flox/flox^ -propofol group (Fig. 4D–F) during the probe trial. In the Y maze test, the p16^INK4a^-CKO-propofol group exhibited a significantly higher rate of spontaneous alternations than those in the p16^INK4a flox/flox^ -propofol group, indicating a better working memory (Fig. 4G, H). In the testing phase of NOR test, the p16^INK4a^-CKO-propofol group showed an increased RI than those in the p16^INK4a flox/flox^ -propofol group (Fig. 4J–K). Additionally, significantly less SA-β-gal staining in the hippocampus of p16^INK4a^-CKO-propofol mice was observed than that in age-matched p16^INK4a flox/flox^ -propofol mice (Fig. 3J, K). These results show that neuronal-specific knockout of p16^INK4a^ prevents propofol-induced neuronal senescence and cognitive decline.

**Fig. 5:**
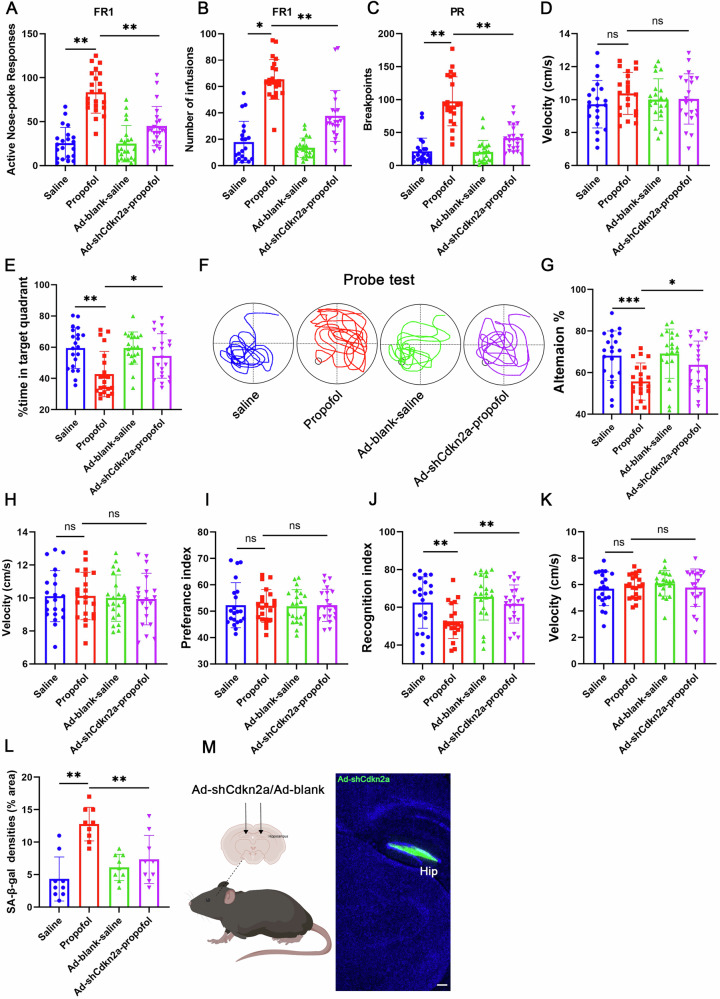
The inhibition of p16^INK4a^ prevents propofol-induced neuronal senescence and cognitive decline in Ad-shCdkn2a mice. **A** Propofol-reinforced active nose-pokes and (**B**) Propofol-reinforced infusions under FR1 reinforcement. n = 21 mice/group. **C** The breakpoint number under PR reinforcement. n = 21 mice/group. **D** average velocity during the probe test for mice in the MWM test. **E** percentage of time spent swimming in the target quadrant, (**F**) Swim paths. **G** Alternation of arms in the spontaneous alternation test of mice in the Y-maze test and (**H**) average velocity during the spontaneous alternation test of mice in the Y-maze test. Novel object recognition test (NOR). Preference index (**I**), recognition index (**J**), and (**K**) average velocity during the NOR test. **L** Quantification of SA-β-gal positive cells in the indicated HT22 cells. **M** A Schematic representation of viral injections into the hippocampus and representative IHC images of DAPI (blue) and GFP (green, viral marker) 6 weeks after viral injections. Scale bars represent 250 µm. n = 21 mice/group. For all figures: Values mean ± SD. Statistical significance was assessed using two-way ANOVA with post hoc Tukey’s multiple comparisons test. *P < 0.05, **P < 0.01, and ***P < 0.001.

Memory performance was assessed after 4 weeks post stereotaxic injection. Consistently, after knockdowning p16^INK4a^ expression in hippocampus by stereotaxic injection of pADV-U6-shCdkn2a-CMV-EGFP (Ad-shCdkn2a), we found increased cognitive function (Fig. 5A–K), and less SA-β-gal staining compared with mice stereotaxic injected pADV-U6-blank-CMV-EGFP (Ad-blank) in hippocampus (Fig. 5L). Taken together, these data show p16^INK4a^ inhibition prevents senescence and cognitive decline induced by propofol. These loss-of-function studies definitively position p16^INK4a^ as the critical molecular switch converting transient propofol exposure into irreversible senescence, while demonstrating therapeutic potential of p16^INK4a^-targeted gene editing even after neurosenescence initiation.

### ADAR1/SIRT1 axis mediates p16^INK4a^-dependent senescence in propofol-treated neurons

**Fig. 6:**
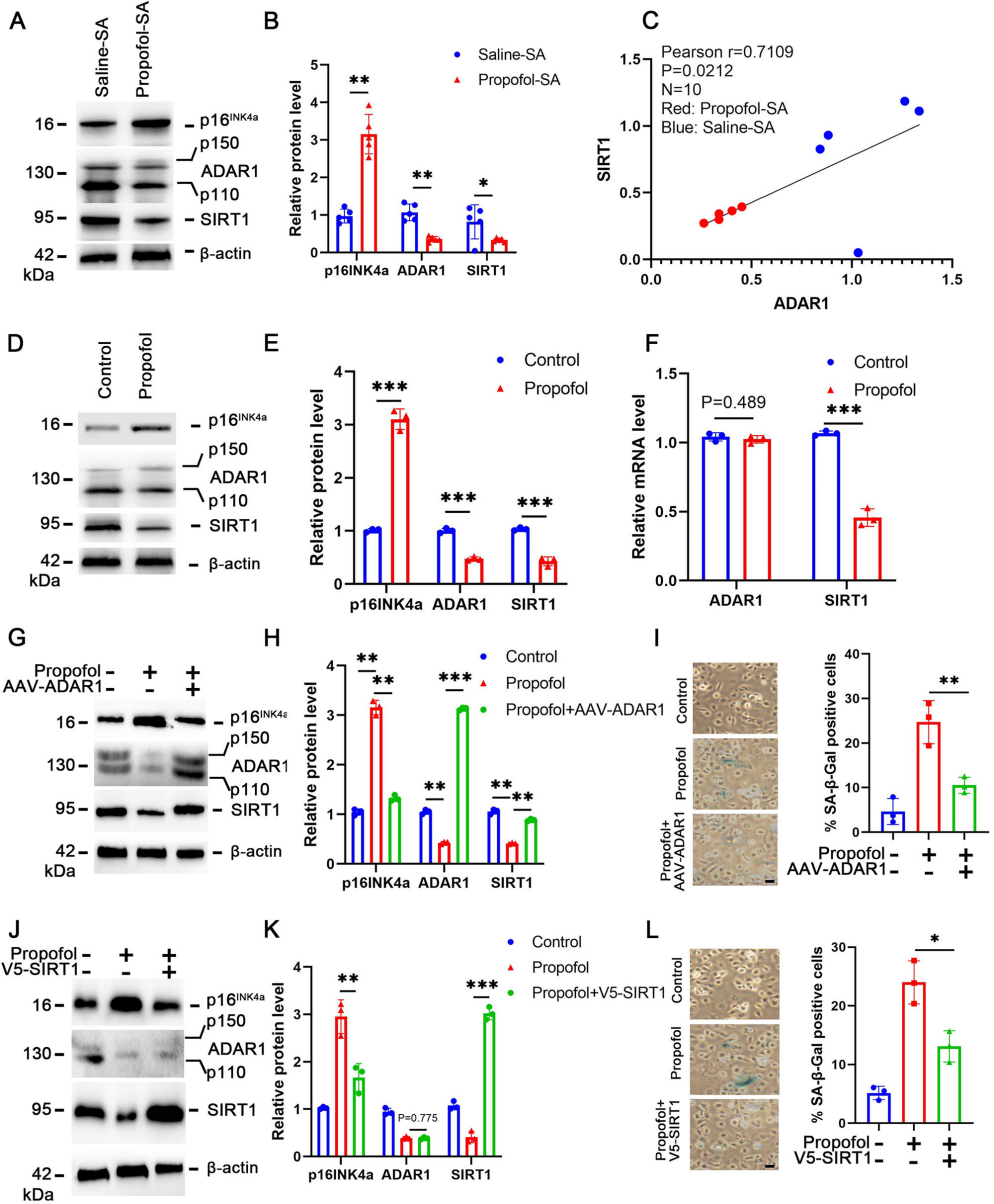
Propofol induces p16^INK4a^-dependent senescence through ADAR1/SIRT1 pathway. **A** Expression of p16^INK4a^, ADAR1 and SIRT1 were determined by Western blotting analysis in the hippocampus harvested from Saline-SA and Propofol-SA mice. **B** The relative expression of p16^INK4a^, ADAR1 and SIRT1 by normalizing against β-actin expression. **C** Correlation between ADAR1 and SIRT1 protein expression as determined by a two-sided Pearson correlation analysis. **D** Expression of p16^INK4a^, ADAR1 and SIRT1 were determined by Western blotting analysis in HT22 cells treated with propofol or saline (Control). **E** The relative expression of p16^INK4a^, ADAR1 and SIRT1 by normalizing against β-actin expression. **F** qRT-PCR analysis was used to detect the mRNA level of ADAR1 and SIRT1. **G** Expression of p16^INK4a^, ADAR1 and SIRT1 were determined by Western blotting analysis in the indicated HT22 cells. **H** The relative expression of p16^INK4a^, ADAR1 and SIRT1 by normalizing against β-actin expression. **I** Representative SA-β-gal staining and quantification of SA-β-gal positive cells in the indicated HT22 cells. **J** Expression of p16^INK4a^, ADAR1 and SIRT1 were determined by Western blotting analysis in the indicated HT22 cells. **K** The relative expression of p16^INK4a^, ADAR1 and SIRT1 by normalizing against β-actin expression. **L** Representative SA-β-gal staining and quantification of SA-β-gal positive cells in the indicated HT22 cells. All data are representative of at least three times. For all figures: Values mean ± SD. Statistical significance was assessed using two-way ANOVA with post hoc Tukey’s multiple comparisons test. *P < 0.05, **P < 0.01, and ***P < 0.001.

Mechanistic interrogation revealed that propofol hijacks the ADAR1-SIRT1 regulatory axis to drive p16^INK4a^-dependent senescence. The ADAR1/SIRT1 pathway has been proved involved in regulating p16^INK4a^ expression in senescence. In the present study, we found that the protein level of ADAR1 was significantly decreased in the hippocampus of propofol-SA mice compared with saline-SA mice (Fig. 6A, B). Consistently, the protein level of SIRT1 was significantly decreased in the hippocampus of propofol-SA mice compared with saline-SA mice (Fig. 6A, B). Moreover, we found a positive correlation between ADAR1 and SIRT1 expression in hippocampus of propofol-SA mice (Fig. 6C). We further confirm these results in vitro and found that SIRT1 downregulation at both protein and mRNA levels and ADAR1 downregulation only at protein level upon propofol treatment (Fig. 6D–F). Propofol failed to downregulate SIRT1 expression, upregulate p16^INK4a^ expression and induce senescence when rescue the expression of ADAR1 by AAV (Fig. 6G–I). Notably, ectopic SIRT1 also overcomed senescence and suppressed p16^INK4a^ upregulation induced by propofol (Fig. 6J–L).

Emerging evidence indicates that ADAR1 interacts with human antigen R (HuR), an RNA-binding protein regulating post-transcriptional stability whose binding to target RNAs is ADAR1-dependent. To investigate whether propofol modulates the ADAR1/SIRT1 axis through HuR-mediated regulation of SIRT1 mRNA, we conducted RNA immunoprecipitation (RIP) assays with anti-HuR antibodies. Our data demonstrated that propofol exposure markedly reduced HuR-SIRT1 mRNA complex formation (Fig. S1A). Notably, ADAR1 overexpression abolished the propofol-induced reduction in HuR/SIRT1 mRNA interactions (Fig. S1B), establishing that propofol-mediated ADAR1 downregulation drives SIRT1 mRNA destabilization through impaired HuR binding capacity.

Additionally, our data revealed that propofol exposure markedly enhanced the binding affinity between eukaryotic translation initiation factor 3 subunit A (EIF3a) and p16^INK4a^ mRNA (Fig. S1C), which correlated with concomitant increases in p16^INK4a^ translational efficiency as evidenced by polysome profiling assays (Fig. S1D). Notably, SIRT1 overexpression abolished the propofol-induced EIF3a-p16^INK4a^ mRNA interaction (Fig. S1E). Mechanistically, propofol-induced ADAR1 downregulation drives cellular senescence through a multistep regulatory cascade: (1) Reduced ADAR1 expression impairs HuR-mediated stabilization of SIRT1 mRNA, leading to accelerated SIRT1 transcript degradation; (2) Subsequent SIRT1 protein depletion activates EIF3a-dependent translational machinery; (3) Enhanced EIF3a-p16^INK4a^ mRNA interaction promotes selective translation of senescence-associated markers. These results suggest that ADAR1/SIRT1 pathway involved in propofol induced p16INK4a-dependent senescence.

### Autophagy activation promotes ADAR1 degradation following propofol exposure

**Fig. 7:**
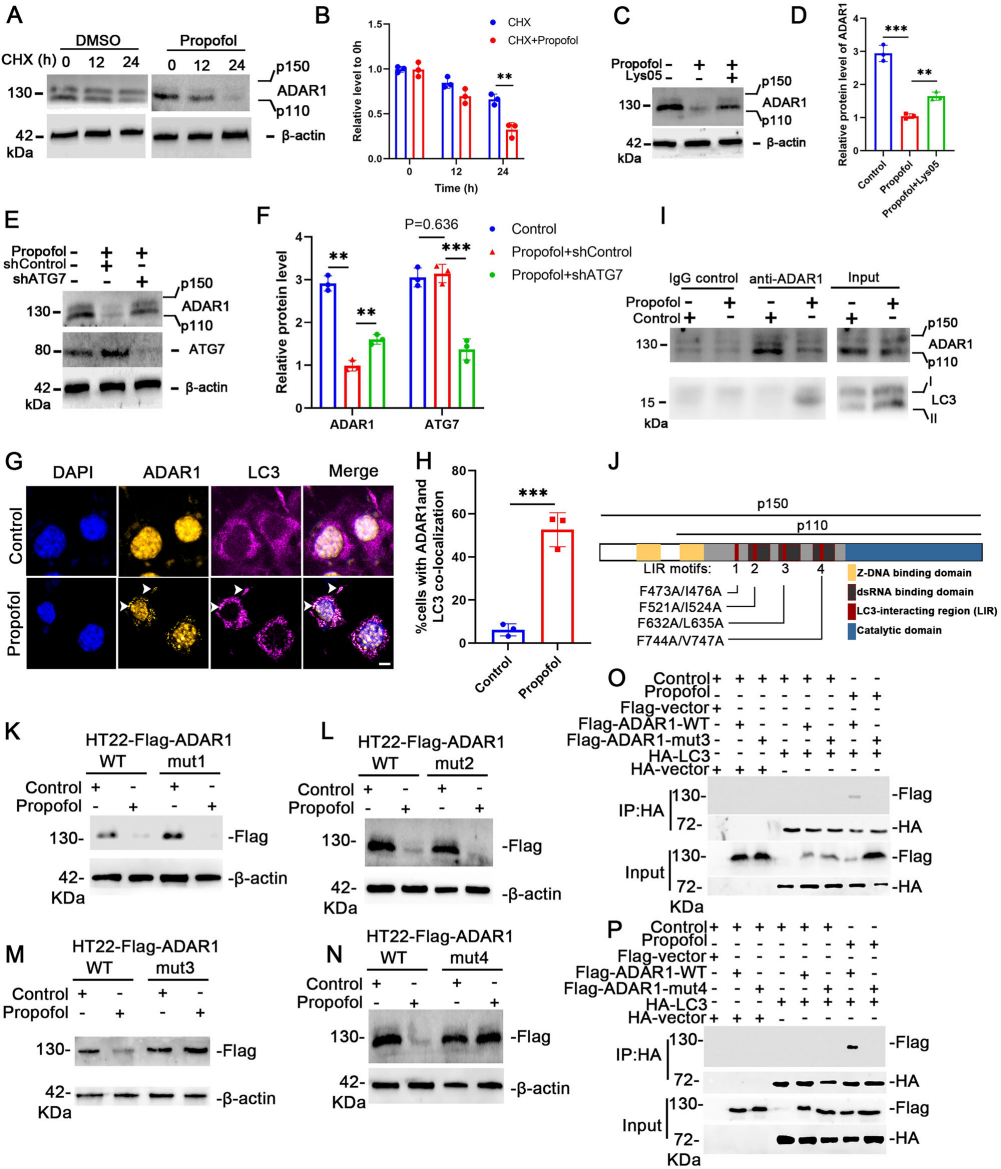
Propofol induces ADAR1 degradation by activating autophagy. **A** Western blotting analysis was used to detect ADAR1 expression in HT22 cells treated with CHX + DMSO or CHX+ Propofol for 0, 12, 24 h. **B** Relative protein level of ADAR1 to 0 h. **C** Western blotting analysis was used to detect the protein levels of ADAR1 after different treatments. **D** Relative protein level of ADAR1. **E** Western blotting analysis was used to detect the protein levels of ADAR1 and ATG7 after different treatments. **F** Relative protein level of ADAR1 and ATG7. **I** Co-immunoprecipitation analysis between ADAR1 and LC3 in cytoplasmic extracts from the indicated HT22 cells. **G** Representative confocal microscopy images illustrating endogenous ADAR1 and LC3 immunostaining in control and propofol-treated groups. Scale bars: 10 μm. **H** Quantification of cells exhibiting co-localization between cytoplasmic ADAR1 puncta and LC3-positive autophagosomes, with representative overlapping signals highlighted by arrowheads. **J** Schematic diagram of the ADAR1 protein structure, annotated to indicate the positions of four conserved LC3-interacting region (LIR) motifs. **K**–**N** HT22-Flag-ADAR1 cells or HT22 cells stably expressed the indicated Flag-ADAR1 mutants were treated with propofol for 24 h. The cell lysates were subjected to Western blotting using the indicated antibodies. **O**, **P** Flag-tagged ADAR1, ADAR1-mut3/mut4, and HA-tagged LC3 expression plasmids were co-transfected to HEK293T cells for 24 h. The cell lysates were subjected to IP with an anti-HA antibody and then analyzed by Western blotting. All data are representative of at least three times. For all figures: Values mean ± SD. Statistical significance was assessed using two-way ANOVA with post hoc Tukey’s multiple comparisons test. *P < 0.05, **P < 0.01, and ***P < 0.001.

We explored the underlying mechanism for the downregulation of ADAR1 induced by propofol. The mRNA level of ADAR1 was not downregulated in propofol induced senescence (Fig. 6F), and the ADAR1 degradation rate was significantly accelerated upon propofol treatment, indicating a post-transcriptional downregulation of ADAR1 by propofol (Fig. 7A, B). The autophagy pathway has been proved to involve in the degradation of ADAR1. Indeed, applying the autophagy-lysosome pathway inhibitor Lys05 into propofol-treated cells could rescue the protein downregulation of ADAR1 (Fig. 7C, D). Likewise, knocking down the upstream autophagy regulator ATG7 could impair the protein downregulation of ADAR1 in propofol-treated cells (Fig. 7E, F).

To determine whether ADAR1 is an autophagy substrate in propofol induced neuronal senescence, we examined the interaction between LC3 and ADAR1. Firstly, LC3 and ADAR1 co-localization was observed in the cytoplasm of propofol-treated cells (Fig. 7G, H). The co-immunoprecipitation analysis results showed enhanced interaction between LC3 and ADAR1 in propofol-treated cells (Fig. 7I). Indeed, ADAR1 harbored four LC3-interacting region (LIR) motifs that could be potentially recognized by LC3 (Fig. 7J). We generated a line of HT22 cells stably expressing FlagADAR1 (HT22-Flag-ADAR1) and found that LIR3 and LIR4 ADAR1 mutants, which were marked as F632A/L635A (mut3) and F744A/V747A (mut4), showed significant resistance to propofol-induced degradation (Fig. 7K–N), and these two mutants failed to bind to LC3 (Fig. 7O, P). These results disclosed that propofol induces ADAR1 degradation by activating the autophagy pathway. These results support the autophagy-lysosome pathway of ADAR1 degradation during propofol-induced senescence.

### Inhibition of autophagy reverses propofol-induced neurosenescence and addictive behaviors

**Fig. 8:**
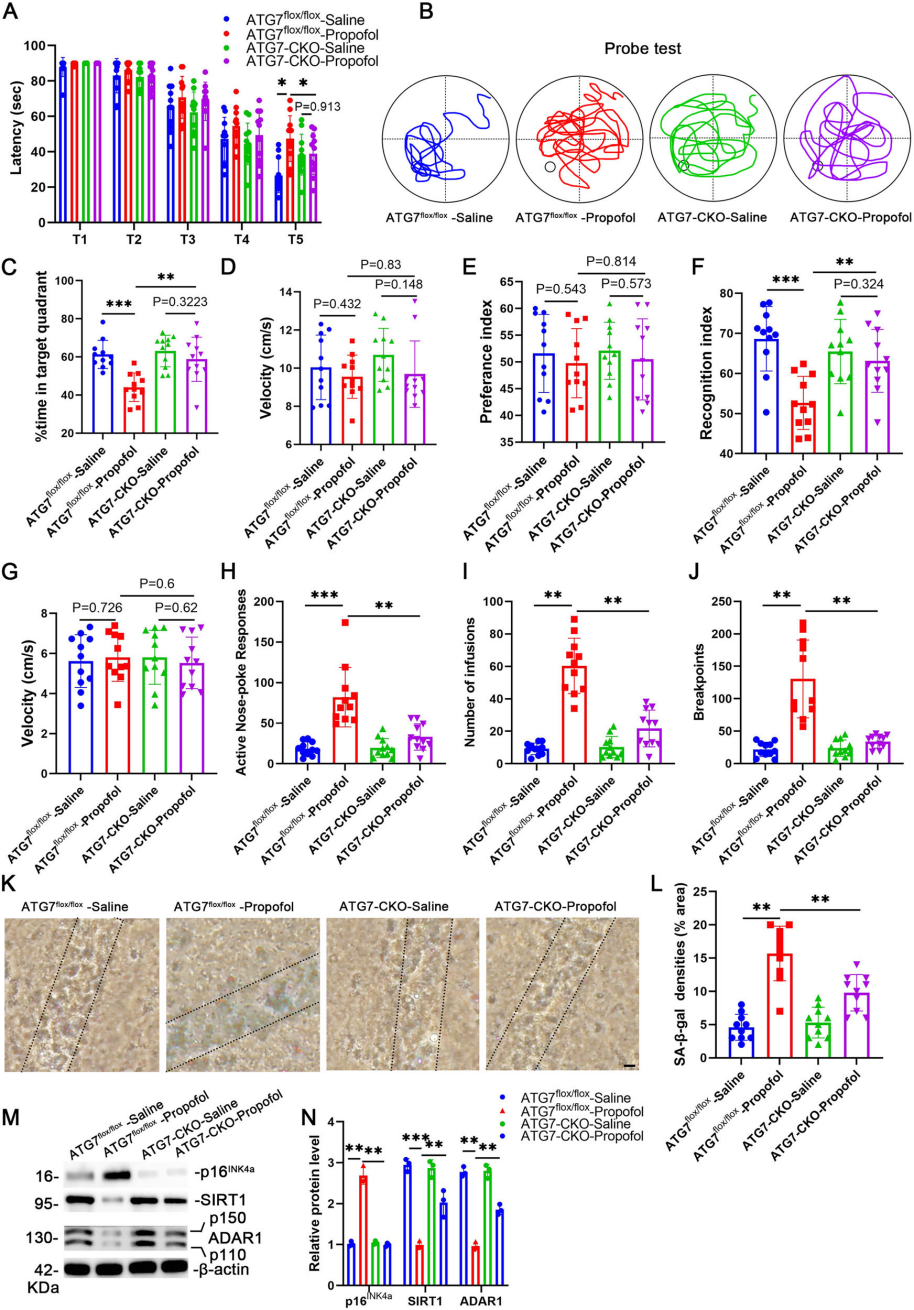
Inhibition of autophagy ameliorated propofol-induced neuronal senescence, cognitive decline, propofol consumption and motivation. **A** Escape latencies (s) of ATG7^flox/flox^-saline, ATG7^flox/flox^-Propofol, ATG7-CKO-saline, and ATG7-CKO- Propofol mice in the MWM test. **B** Swim paths, **C** percentage of time spent swimming in the target quadrant, and (**D**) average velocity during the probe test for mice in the MWM test. Novel object recognition test (NOR). Preference index (**E**), recognition index (**F**), and (**G**) average velocity during the NOR test. **H** Propofol-reinforced active nose-pokes and (**I**) Propofol-reinforced infusions under FR1 reinforcement. n = 8 mice/group. **J** The breakpoint number under PR reinforcement. n = 11 mice/group. **K** Representative SA-β-gal staining in hippocampus. **L** Quantification of SA-β-gal staining densities in A. n = 11 mice/group. Scale bars = 50 μm. **M** Western blotting analysis was used to detect specific proteins in hippocampus. n = 3 mice/group. **N** Relative protein levels of p16^INK4a^, ADAR1 and SIRT1. For all figures: Values mean ± SD. *P < 0.05, **P < 0.01, and ***P < 0.001.

We next evaluated whether autophagy pathways are responsible for propofol-induced neuronal senescence, cognitive decline, propofol consumption, and motivation. The ATG7^flox/flox^ mice were bred with the CaMKIIa-driven Cre recombinase transgenic mice, and a neuronal-specific ATG7-deficient mouse model (ATG7-CKO mice) was generated. After 14 days of SA, the ATG7-CKO mice self-administered with propofol (ATG7-CKO-propofol) and ATG7^flox/flox^ mice self-administered with propofol (ATG7^flox/flox^ -propofol) were subjected to behavioral tests related to cognitive function. In the MWM test, the ATG7-CKO-propofol group spent significantly less time in escape latency during the last training day than the ATG7^flox/flox^ -propofol group (Fig. 8A). During the probe trial, the ATG7-CKO-propofol group spent less time exploring the target quadrant than the ATG7^flox/flox^ -propofol group (Fig. 8B–D). In the testing phase of NOR test, the ATG7-CKO-propofol group showed an increased RI than those in the ATG7^flox/flox^ -propofol group (Fig. 8E–G). Additionally, significantly less SA-β-gal staining (Fig. 8K, L), decreased p16^INK4a^ expression, increased ADAR1 and SIRT1 expression in the hippocampus of ATG7-CKO-propofol mice were observed than that in age-matched ATG7^flox/flox^ -propofol mice (Fig. 8M, N). These results show that neuronal-specific knockout of ATG7 prevents propofol-induced neuronal senescence and cognitive decline.

Furthermore, under the FR1 reinforcement schedule, the ATG7-CKO-propofol mice group significantly reduced propofol active nose-poke responses (Fig. 8H) and propofol infusions (Fig. 8I) compared with the ATG7^flox/flox^ -propofol mice group. Under the PR reinforcement schedule, the ATG7-CKO-propofol mice group significantly reduced breakpoints compared with the ATG7^flox/flox^ -propofol mice group (Fig. 8J). These results suggested that propofol consumption and motivation were decreased by autophagy inhibition. These findings position pharmacological autophagy modulation as a promising therapeutic axis, capable of reversing established neurosenescence while disrupting the self-perpetuating cycle of propofol addiction through proteostasis restoration.

## Discussion

Cognitive decline induced by addiction, such as the dysfunction of decision-making, attention deficit and impulsivity, has been reported previously. However, few studies focused on the general learning and memory decline and underlying mechanism caused by drug abuse. Our study aimed to investigate whether propofol addiction co-opts neuronal senescence pathways. In direct support of this, we demonstrated propofol addiction caused general learning and memory decline in mice. p16^INK4a^ dependent cellular senescence induced by propofol are observed in vivo and in vitro. Additionally, autophagy-mediated ADAR1 degradation and ADAR1/SIRT1 pathway are essential for propofol-induced senescence and cognitive decline. Neuronal-specific and systemic autophagy blockage in mouse models ameliorated propofol-induced senescence, cognitive decline, propofol consumption, and motivation. Taken together, the general learning and memory deficits are essential pathological features of propofol addiction and focusing on autophagy and cognitive decline induced by propofol may serve as key targets for developing addiction therapies.

As an anesthetic, there has been controversy over propofol causing cognitive impairment and two main theories exist. (1) Propofol amplifies the cognitive impairment of animals via age or specific transgenes. Elderly patients suffer from postoperative cognitive dysfunction after surgery^21^. Pregnant rats showed cognitive disorders after anesthetized with propofol^22^. (2) Propofol has minimal effects on cognitive function, and even results in better cognitive outcomes^23^. 3xTgAD mice received vehicle or propofol treatment without surgery and the MWM results showed no significant differences between these two groups in escape latencies^24^. 180 elderly patients were randomly divided into slow injections of propofol, medium injection of propofol and fast injection of propofol. Mini-Mental State Examination and Montreal Cognitive Assessment were used to measure cognitive function before and after propofol administration. The results showed no statistically significant differences between the three groups^25^.

Propofol addiction induced cognitive decline is apart from the two conditions, because of the high dosage and long duration administration of drug. Our research is the first to prove cognitive decline induced by propofol addiction using self-administration model in mice. We have identified general learning and memory deficits in propofol-SA mice (MWM, Y maze, and NOR tests), indicating that propofol impairs cognitive function. An interesting aspect of our propofol SA model is the required dose (2 mg/kg/infusion) to maintain reliable self-administration. When placed in the broader context of addiction research, this dose is orders of magnitude higher than the reinforcing doses of classic stimulants like methamphetamine. This is not to directly compare their addictive potential, but rather to highlight a key pharmacological difference: propofol, as a sedative, may require a higher threshold dose to produce a detectable reinforcing signal within the context of an operant behavior paradigm, possibly due to its sedating effects competing with operant responding. Our study, therefore, not only provides new evidence that cognitive deficits are a core pathological feature of propofol addiction but also underscores the unique behavioral pharmacology of this sedative-hypnotic drug.

The overexpression of p16^INK4a^ is often used as a specific and unique marker for senescence^26^. It is of high significance to understand how p16^INK4a^ expression is regulated in cellular senescence. The transcriptional activation of p16^INK4a^ has been reported extensively in senescent cells, and many p16^INK4a^ inducers, from accessibility of promoter to stability of protein, have been described as epigenetic regulators at the transcriptional levels^27,28^. Herein, we demonstrate that the translational level of p16^INK4a^ is regulated during propofol induced senescence. Autophagy, which is a lysosomal degradation pathway, routes cytoplasmic damaged subcellular structure and protein aggregates to lysosomes for degradation and maintains homeostasis. In the present study, we demonstrate autophagy promotes the degradation of ADAR1 upon propofol treatment, followed by the downregulation of ADAR1 controlled SIRT1, resulting in the activation of p16^INK4a^ dependent cellular senescence. Previous studies support our result that autophagy mediates SIRT1 and lamin B1degradation to contribute to senescence^29,30^.

Our data demonstrates that the downregulation of ADAR1 protein is both necessary and sufficient for the upregulation of p16^INK4a^ protein and the ensuing senescence phenotype. Notably, this effect occurred without a significant change in p16^INK4a^ mRNA levels (Fig. S1E), pointing to a post-transcriptional regulatory mechanism. While ADAR1 is best known for its RNA-editing function, recent studies have described editing-independent roles for ADAR1 in mediating protein-protein interactions and transcript stability^14^. Therefore, we propose that the observed regulation of p16^INK4a^ by ADAR1 may represent a novel, editing-independent function, although future studies using editing-deficient ADAR1 mutants are required to formally exclude a contribution of its enzymatic activity. Decreased expression of ADAR1 was observed in hippocampus tissues obtained from propofol-SA mice compared with those from Saline-SA mice. Additionally, ADAR1 downregulation, neuronal senescence, cognition decline, propofol consumption and motivation can be restored by either neuronal-specific or systemic autophagy blockage. This raises the possibility that inhibiting the autophagy pathway is a critical means to restore propofol induced cognition decline, which may prevent a propofol addiction.

We thank the reviewer for raising the important point regarding the potential involvement of other ADAR family members. The ADAR family has three members, among which ADAR2 and ADAR3 are particularly important in the brain^31,32^. In line with the reviewer’s suggestion, we evaluated the expression of ADAR2 and ADAR3 and found that neither was significantly altered under conditions where ADAR1 was markedly downregulated (Fig. S2). This indicates that the observed senescence phenotype is specifically associated with ADAR1 depletion rather than a general suppression of all ADAR enzymes. The selective downregulation of ADAR1—but not the brain-enriched ADAR2 or ADAR3—suggests a distinct and non-redundant role for ADAR1 in regulating neuronal senescence, possibly through transcript-specific editing or protein-interaction mechanisms that are not compensated by other ADAR isoforms.

There still exist limitations. Firstly, while we provide compelling genetic and pharmacological evidence for the ADAR1-SIRT1-p16^INK4a^ axis, the absence of direct in vivo binding evidence remains a limitation. Secondly, our model primarily examines the initial development of addiction-like behaviors. The long-term persistence of these senescent neurons and their role in relapse remain to be fully elucidated. Finally, the translational potential of targeting senescence in human propofol use disorder requires further investigation in more complex models. Beyond providing a mechanistic link between propofol abuse and cognitive decline, our findings challenge the traditional neurocentric view of addiction. We posit that drug-induced neuronal senescence represents a novel form of cellular allostasis—a persistent, maladaptive state that maintains addictive behavior. This reconceptualization bridges the fields of gerontology and addiction neuroscience, suggesting that therapeutic strategies aimed at rejuvenating the brain’s cellular milieu (‘senolytics’) could be a viable paradigm for treating iatrogenic addictions.

In summary, we report that propofol induces p16^INK4a^ dependent neuronal senescence in hippocampus, which is regulated by autophagy-mediated ADAR1 degradation and ultimately led to cognitive decline. Specifically, the inhibition of autophagy pathway restored propofol-induced neuronal senescence, cognitive decline, propofol consumption, and motivation. This study provides new insight into the neuronal toxicity induced by propofol in hippocampal neurons, contributing to an in-depth understanding of propofol-associated toxicity and cognition dysfunction. Furthermore, our study demonstrated that targeting the cognitive decline induced by propofol is a therapeutic target for addiction.

## Supplementary information

Supplementary Information

Description of Additional Supplementary Files

Supplementary Data

reporting-summary

## Data Availability

The source data for graphs are provided in Supplementary Data. Uncropped/unedited blots are provided in the Supplementary information. All other data are available from the corresponding author on reasonable request.
