# Supplementary material for: Propofol addiction drives neuronal senescence and cognitive decline via autophagy-mediated ADAR1/SIRT1 disruption
Source: Commun Biol. 2025 Dec 22;8:1832. doi: 10.1038/s42003-025-09388-8 (PMC12749188; doi:10.1038/s42003-025-09388-8)
Supplement: Supplementary file 1 — Supplementary Information [file 42003_2025_9388_MOESM1_ESM.pdf]

Supplementary Information

Figure S1.

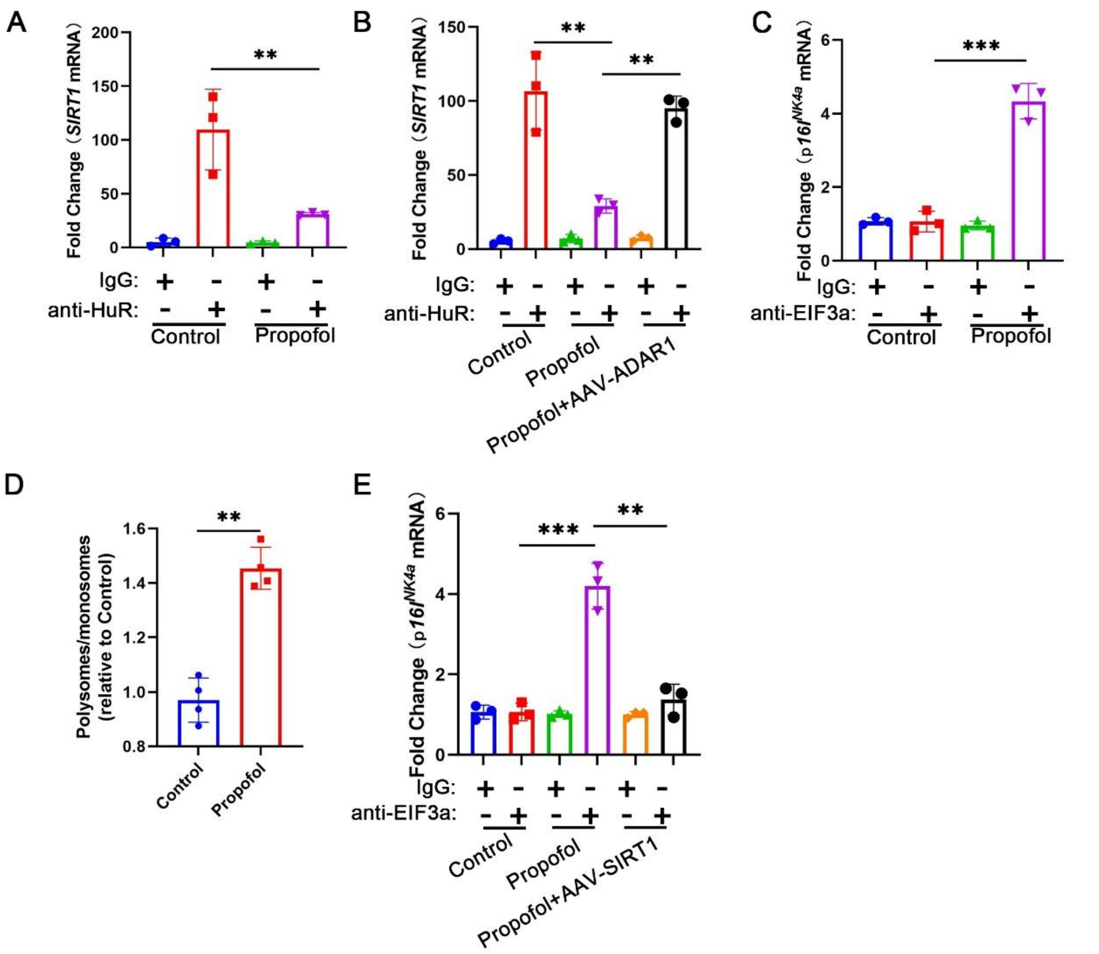

Fig. S1. Propofol-triggered ADAR1 suppression mediates cellular senescence via a multi-step regulatory cascade. (A) RIP (RNA immunoprecipitation) assay using anti-HuR antibody to assess SIRT1 mRNA interactions in saline- or propofol-treated HT22 cells. (B) Comparative RIP analysis of SIRT1 mRNA (anti-HuR) in HT22 cells exposed to saline, propofol, or propofol combined with AAV-ADAR1 overexpression. (C) RIP profiling of p16^{INK4a} mRNA-ELF3a interactions (anti-ELF3a antibody) in saline- vs propofol-treated HT22 cells. (D) Sucrose density gradient-based polysome profiling showing p16^{INK4a} mRNA distribution (polysome/monosome-bound fractions) in propofol-treated vs control HT22 cells, quantified via RT-qPCR. (E) Rescue experiment: RIP

detection of p16^{INK4a} mRNA-ELF3a interactions (anti-ELF3a) in HT22 cells treated with saline, propofol, or propofol + AAV-ADAR1. For all figures: Values mean ± SD. Statistical significance was assessed using two-way ANOVA with post hoc Tukey's multiple comparisons test. *P < 0.05, **P < 0.01, and ***P < 0.001.

Figure S2.

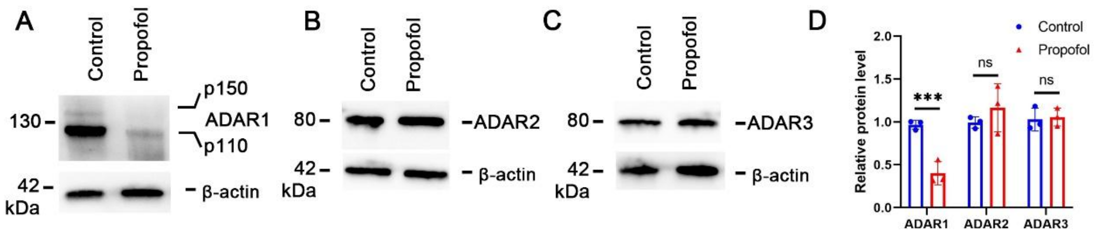

Fig. S2. The expressions of ADAR2 and ADAR3 were not significantly altered under propofol treatment. Expression of ADAR1 (A), ADAR2 (B) and ADAR3 (C) were determined by Western blotting analysis in primary hippocampus neurons with or without propofol treatment. (D) The relative expression of ADAR1, ADAR2 and ADAR3 by normalizing against β-actin expression. Values mean ± SD. Statistical significance was assessed using two-way ANOVA. *P < 0.05, **P < 0.01, and ***P < 0.001.

Supplementary Table 1: List of primers used in this study

**RT-qPCR**	
Sirt1-Forward	5'-TCGCAACTATACCCAGAACATAGACA-3'
Sirt1-Reverse	5'-CTGTTGCAAAGGAACCATGACA-3'
p16-Forward	5'-CACCGAATAGTTACGGTCGG-3'
p16-Reverse	5'-GCACGGGTCGGGTGAGAGTG-3'
ADAR1-total-Forward	5'-AGCACCTTCCATGACCAGATAG-3'
ADAR1-total-Reverse	5'-AGAGAAACCTGATGAAGCCTCTC-3'
18s-Forward	5'-AACTTTCGATGGTAGTCGCCG-3'
18s-Reverse	5'-CCTTGGATGTGGTAGCCGTTT-3'
Gapdh-Forward	5'-GTCTCCTCTGACTTCAACAGCG-3'
Gapdh-Reverse	5'-ACCACCCTGTTGCTGTAGCCAA-3'
**ADAR1 LIR wildtype and mutated sequences subcloned into pBabe-mCherry-GFP plasmid**	
ADAR1-p110-Forward	5'-CATGGATCCATGGCCGAGATCAAGGAGAAAATCT-3'
ADAR1-p150-Forward	5'-CATGGATCCATGAATCCGCGGCAGGGGTATTCCCT-3'
ADAR1-Reverse	5'-CCGCTCGAGCTATACTGGGCAGAGATAAAAGTTC-3'
LIR1-F473A/I476A-Forward	5'-CCAGGTGAGGCACGAGCCGCCATGGAG-3'
LIR1-F473A/I476A-Reverse	5'-CTCCATGGCGGCTCGTGCCTCACCTGG-3'
LIR2-F521A/I524A-Forward	5'-ACCTGTGAGGCAAACATGGCAGAGCAG-3'
LIR2-F521A/I524A-Reverse	5'-CTGCTCTGCCATGTTTGCCTCACAGGT-3'
LIR3-F632A/L635A-Forward	5'-TCCTGCGAAGCACGTCTCGCGTCCAAA-3'
LIR3-F632A/L635A-Reverse	5'-TTTGGACGCGAGACGTGCTTCGCAGGA-3'
LIR4-F744A/V747A-Forward	5'-GCTGCTGAAGCAAAGTTGGCCGACCAG-3'
LIR4-F744A/V747A-Reverse	5'-CTGGTCGGCCAACTTTGCTTCAGCAGC-3'
**HuR RIP-qPCR**	
Sirt1-Forward	5'-CAGCTGCAAAAGCTTCTAGTCT-3'
Sirt1-Reverse	5'-ATGGTCCTAGCTGGGTGTTT-3'
**EIF3A RIP-qPCR**	
p16-Forward	5'-AGATATATGCCTTCCCCCAC-3'
p16-Reverse	5'-CCACATGAATGTGCGCTTAG-3'

Figure 2G

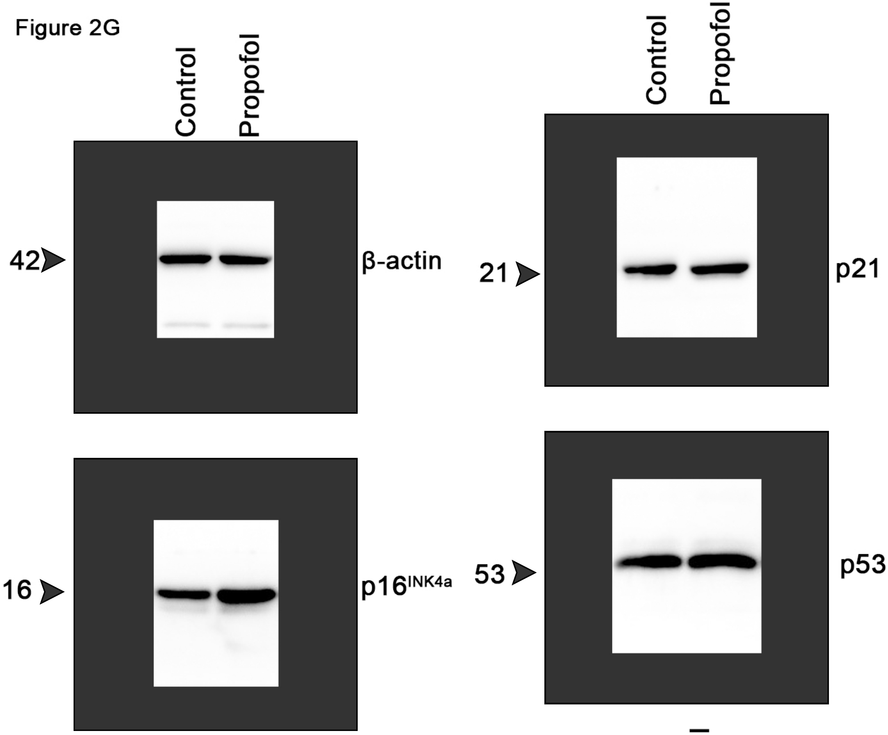

Figure 3D

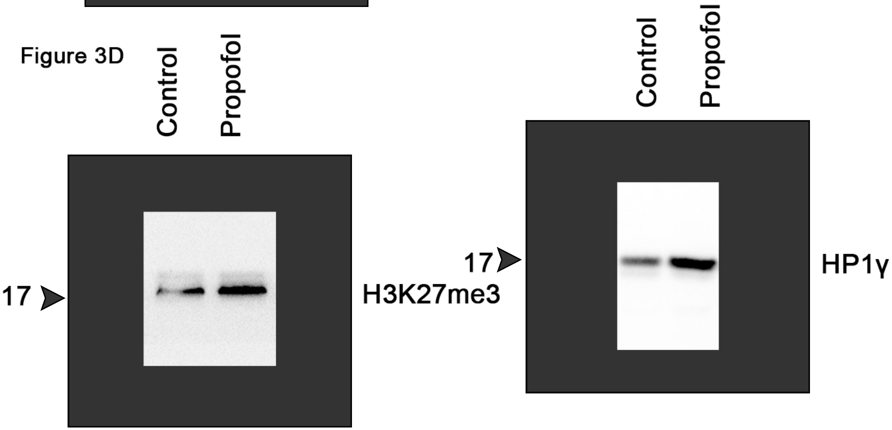

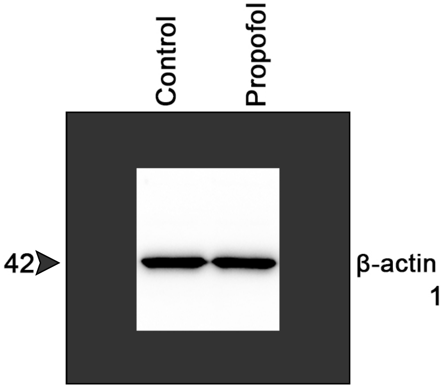
Control
Propofol
42
β-actin

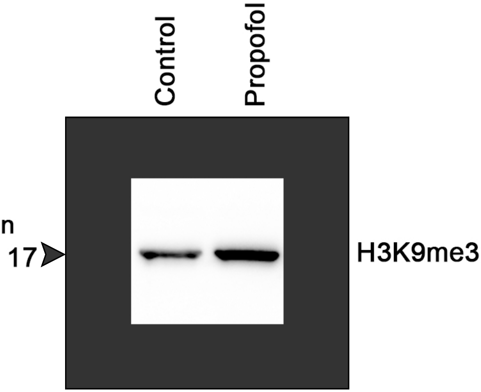
Control
Propofol
17
H3K9me3

Figure 6A

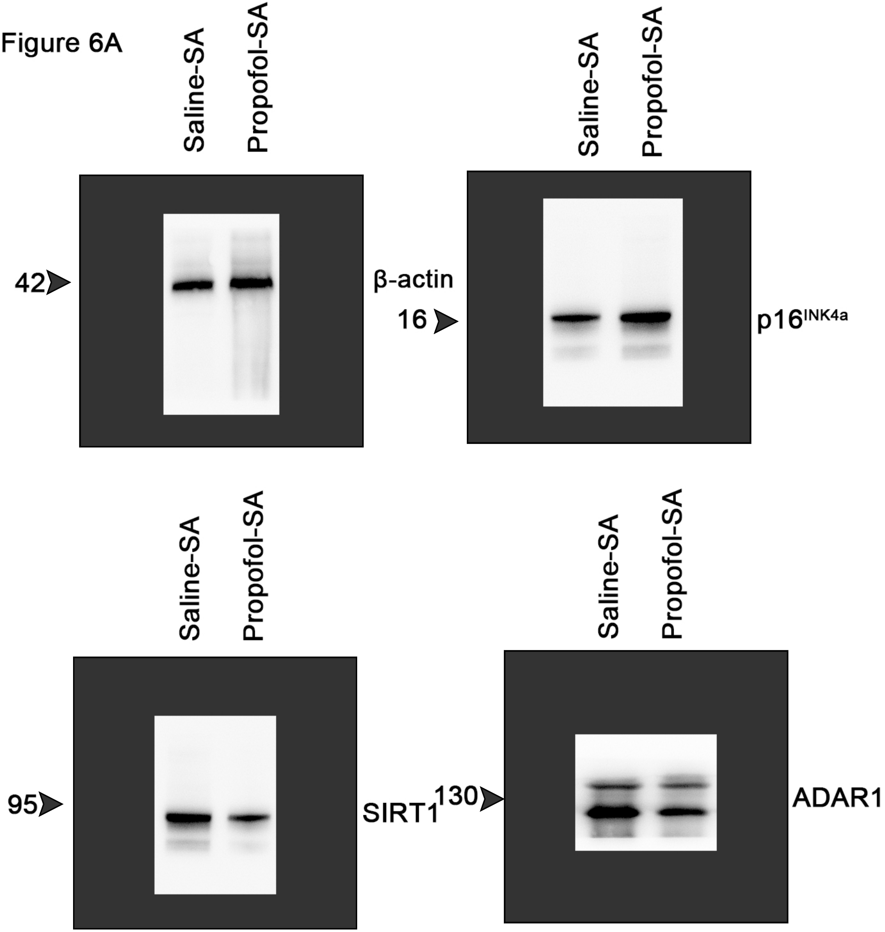

Saline-SA
Propofol-SA
42
β-actin
Saline-SA
Propofol-SA
16
p16INK4a
Saline-SA
Propofol-SA
95
SIRT1
Saline-SA
Propofol-SA
130
ADAR1

Figure 6D

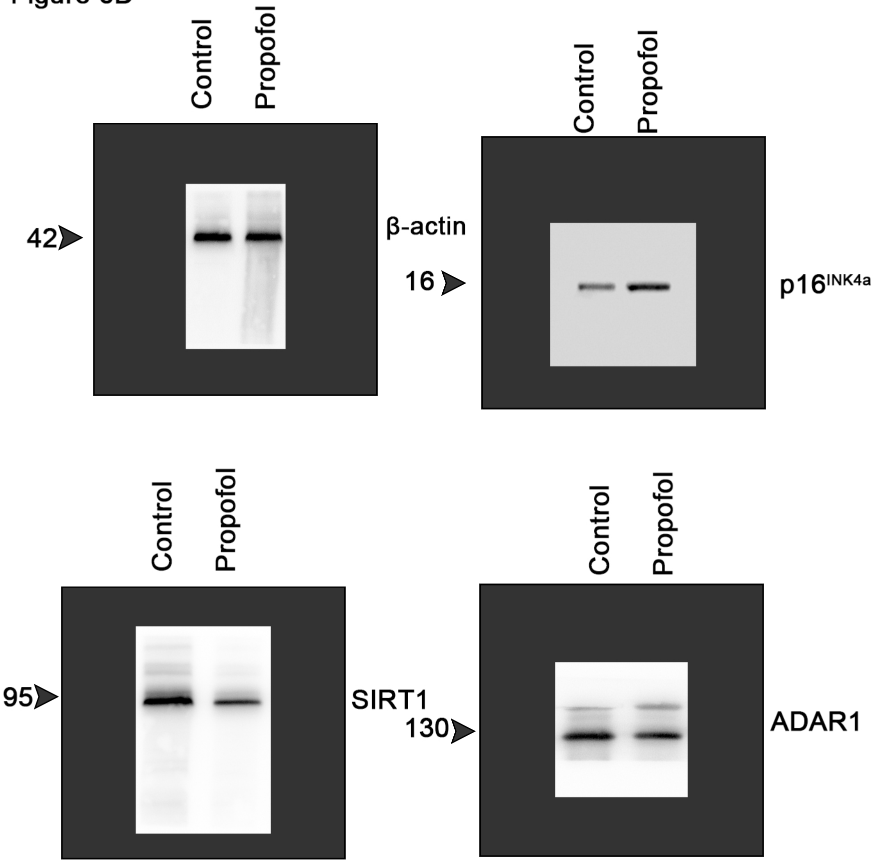
Control
Propofol
42
β-actin
Control
Propofol
16
p16INK4a
Control
Propofol
95
SIRT1
Control
Propofol
130
ADAR1

Figure 6G

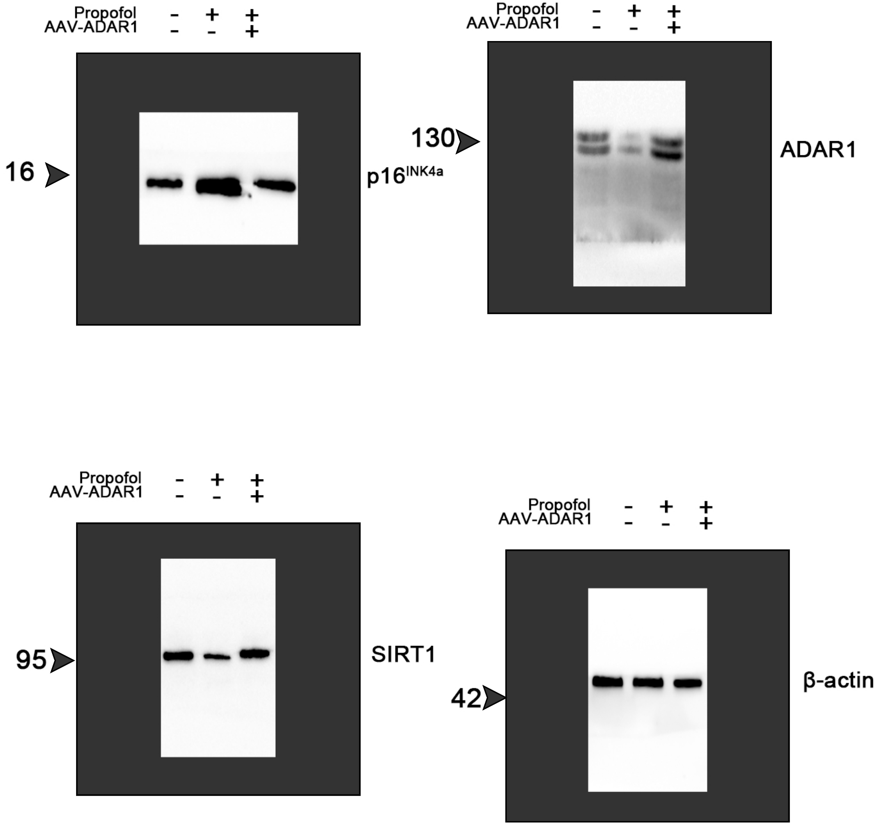

Propofol - + +
AAV-ADAR1 - - +
16
p16INK4a
Propofol - + +
AAV-ADAR1 - - +
130
ADAR1
Propofol - + +
AAV-ADAR1 - - +
95
SIRT1
Propofol - + +
AAV-ADAR1 - - +
42
β-actin

Figure 6J

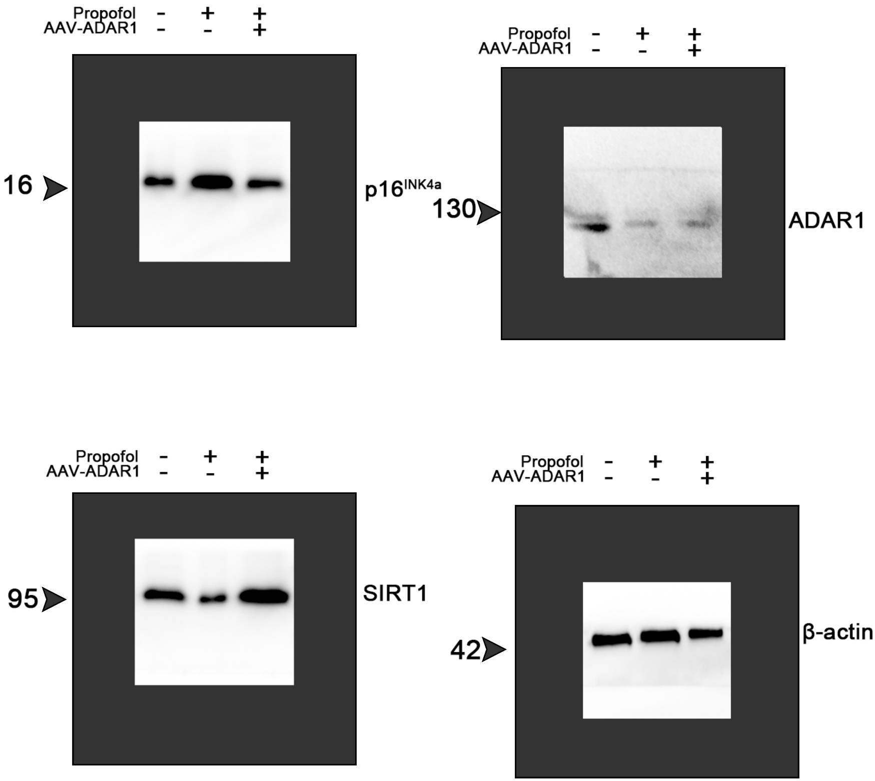

Propofol - + +
AAV-ADAR1 - - +
16
p16INK4a
Propofol - + +
AAV-ADAR1 - - +
130
ADAR1
Propofol - + +
AAV-ADAR1 - - +
95
SIRT1
Propofol - + +
AAV-ADAR1 - - +
42
β-actin

Figure 7A

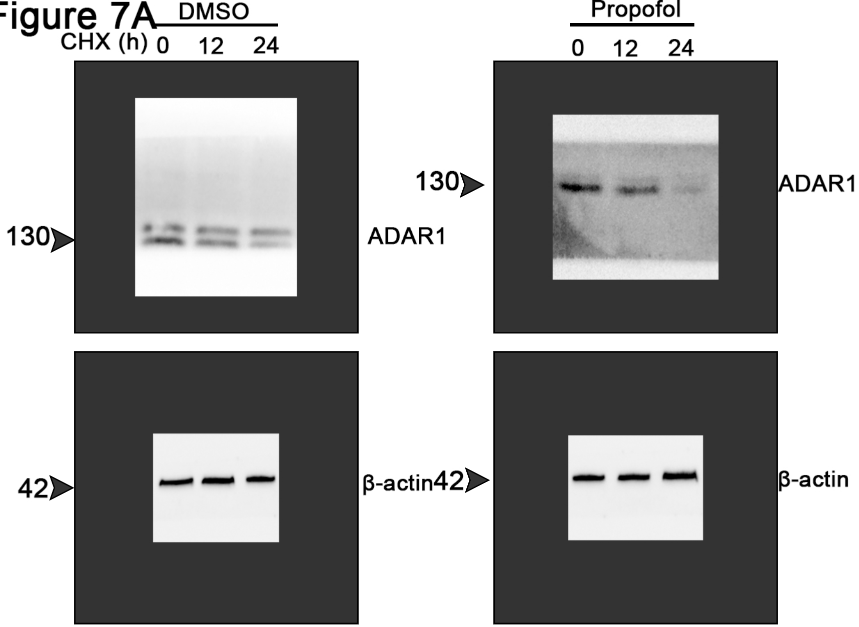

Figure 7C

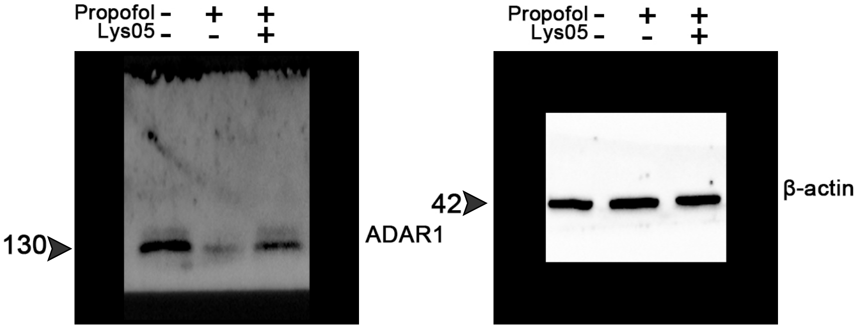

Figure 7E

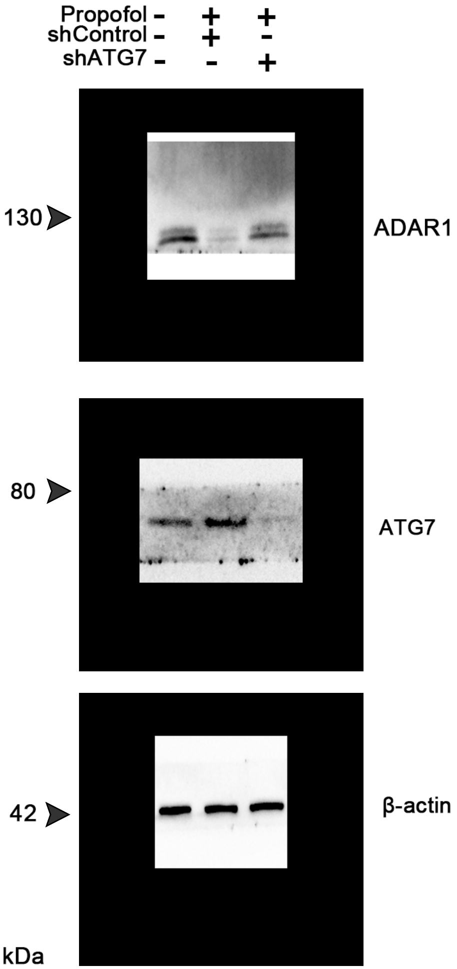

Figure 7I

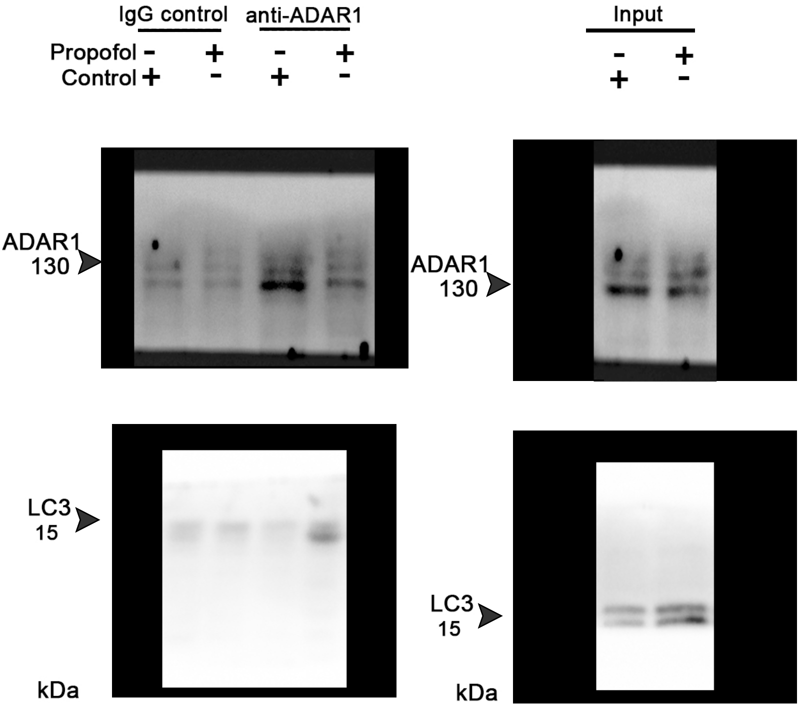
IgG control
anti-ADAR1
Input
Propofol - + - +
Control + - + -
Propofol - +
Control + -
ADAR1
130
ADAR1
130
LC3
15
LC3
15
kDa
kDa

Figure 7K

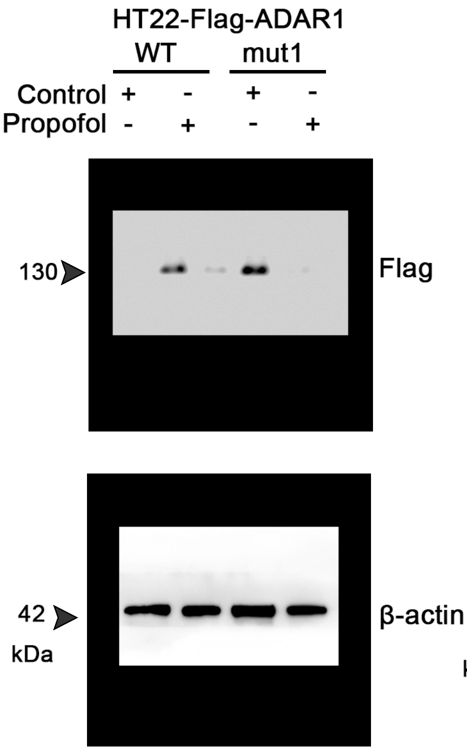

Figure 7L

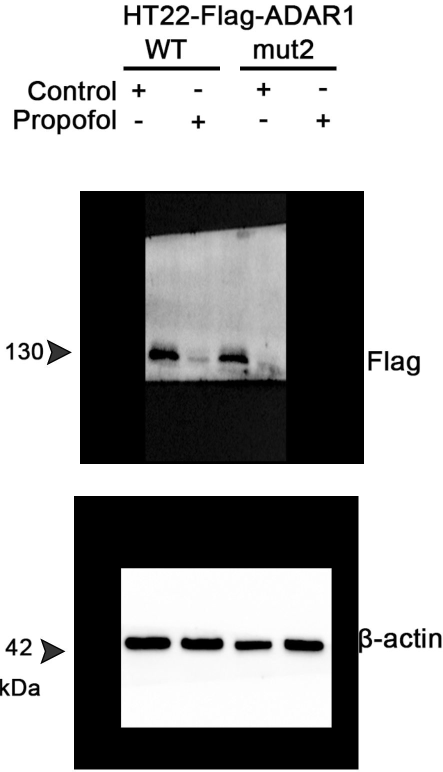

Figure 7M

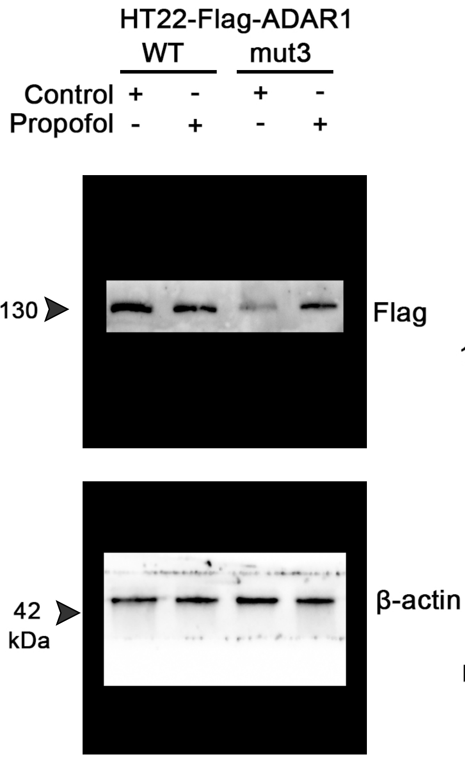

Figure 7N

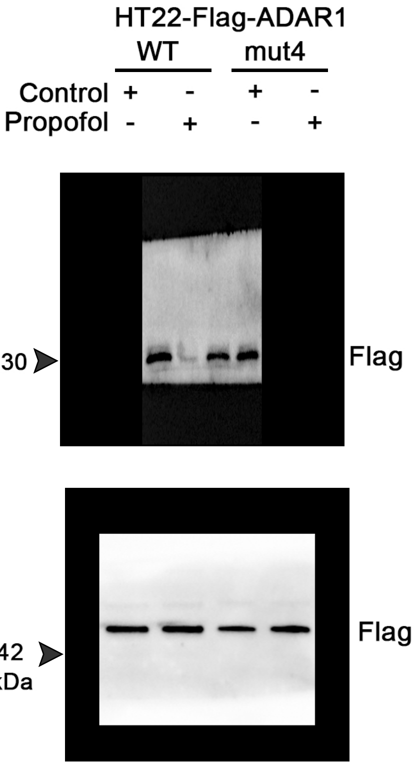

Figure 7O

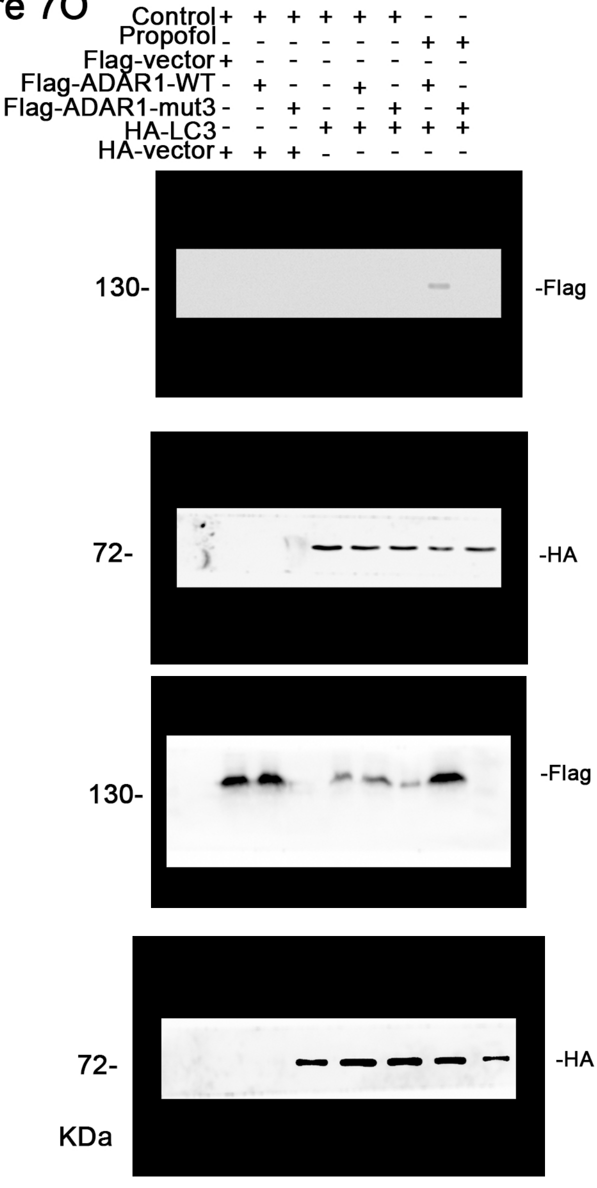
Control + + + + + + - -
Propofol - - - - - - + +
Flag-vector + - - - - - - -
Flag-ADAR1-WT - + - - + - + -
Flag-ADAR1-mut3 - - + - - + - +
HA-LC3 - - - + + + + +
HA-vector + + + - - - - -
130-
-Flag
72-
-HA
130-
-Flag
72-
-HA
KDa

Figure 7P

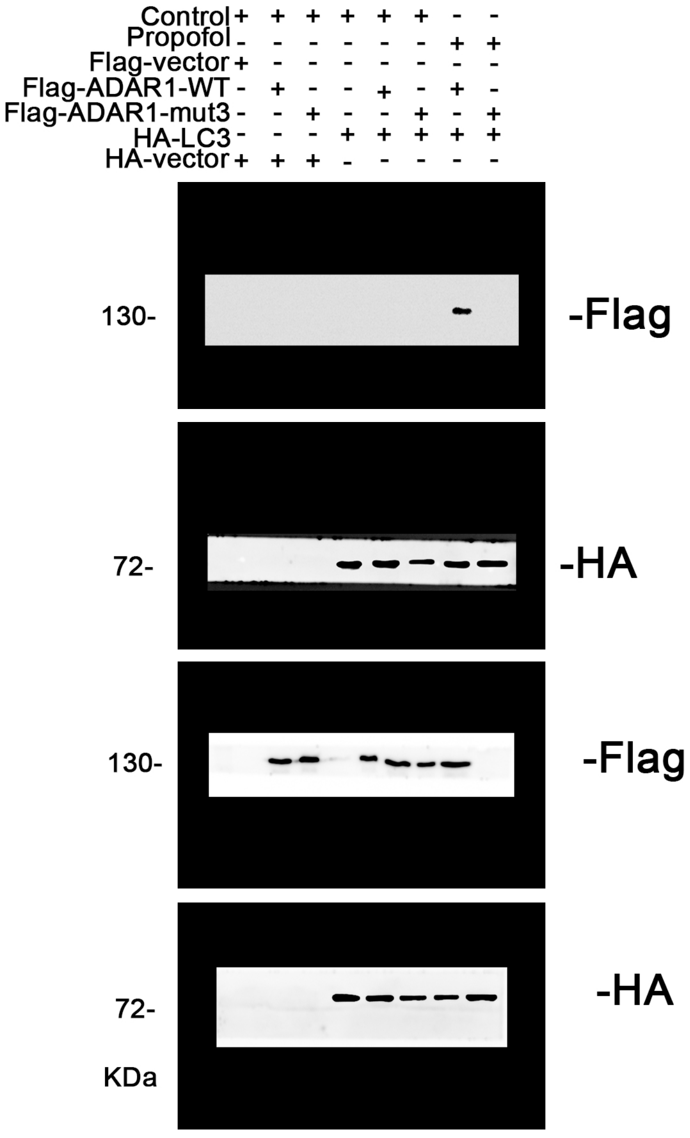
Control + + + + + + - -
Propofol - - - - - - + +
Flag-vector + - - - - - - -
Flag-ADAR1-WT - + - - + - + -
Flag-ADAR1-mut3 - - + - - + - +
HA-LC3 - - - + + + + +
HA-vector + + + - - - - -
130-
-Flag
72-
-HA
130-
-Flag
72-
-HA
KDa

Figure 8M

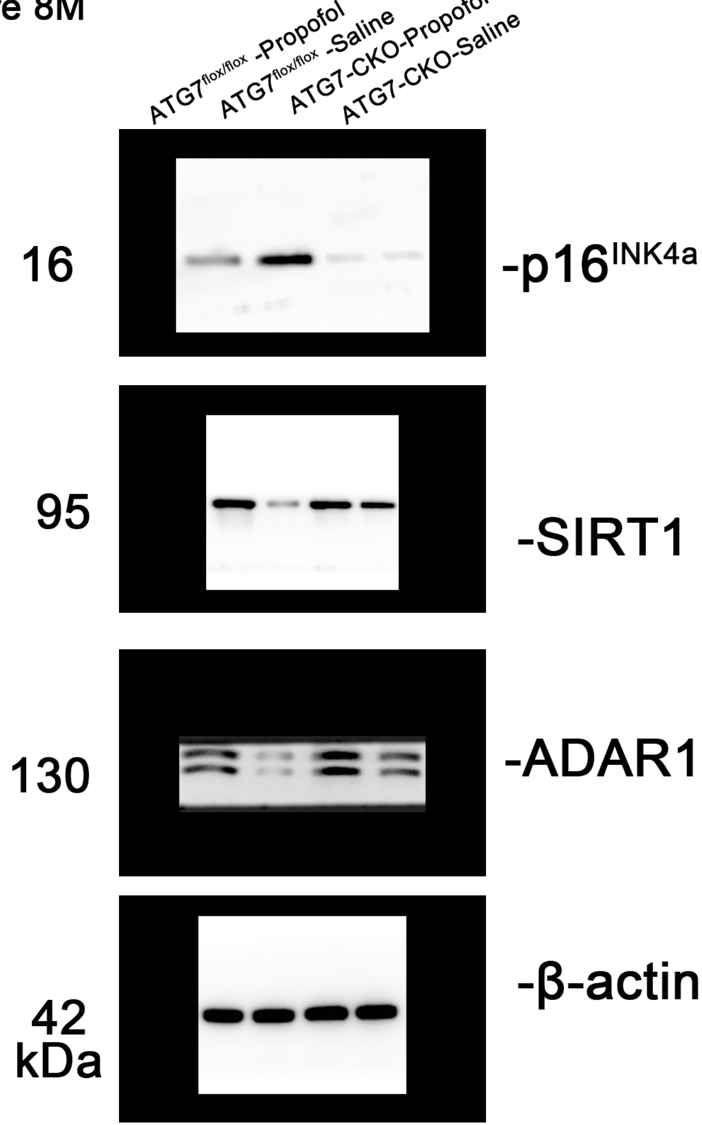
ATG7flox/flox -Propofol
ATG7flox/flox -Saline
ATG7-CKO-Propofol
ATG7-CKO-Saline
16
-p16INK4a
95
-SIRT1
130
-ADAR1
42
kDa
-β-actin

Figure S2

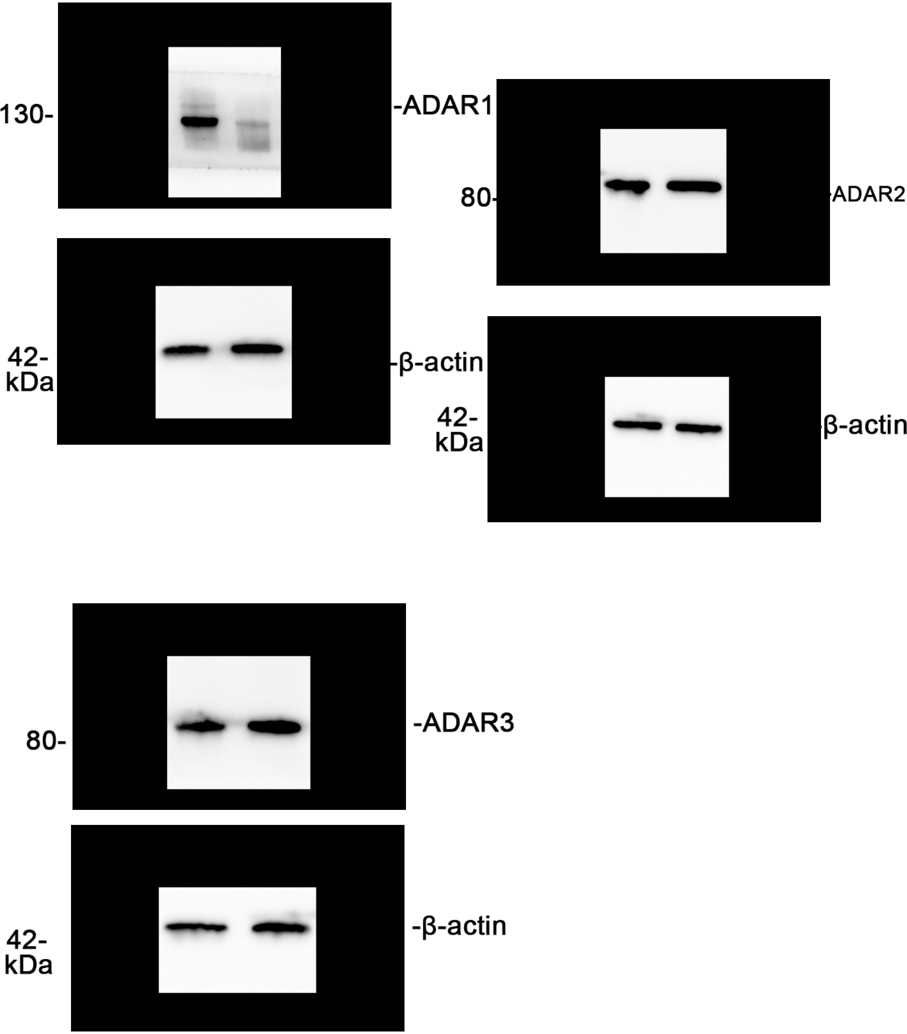

130-
-ADAR1
80-
-ADAR2
42-
kDa
-β-actin
42-
kDa
-β-actin
80-
-ADAR3
42-
kDa
-β-actin
