# Supplementary material for: Propofol addiction drives neuronal senescence and cognitive decline via autophagy-mediated ADAR1/SIRT1 disruption
Source: Commun Biol. 2025 Dec 22;8:1832. doi: 10.1038/s42003-025-09388-8 (PMC12749188; doi:10.1038/s42003-025-09388-8)
Supplement: Supplementary file 2 — Description of Additional Supplementary Files [file 42003_2025_9388_MOESM2_ESM.pdf]

Description of Additional Supplementary Files

File name- Supplementary Data

File description- The source data for graphs are provided in Supplementary Data
