# Supplementary material for: Propofol addiction drives neuronal senescence and cognitive decline via autophagy-mediated ADAR1/SIRT1 disruption
Source: Commun Biol. 2025 Dec 22;8:1832. doi: 10.1038/s42003-025-09388-8 (PMC12749188; doi:10.1038/s42003-025-09388-8)
Supplement: Supplementary file 4 — reporting-summary [file 42003_2025_9388_MOESM4_ESM.pdf]

nature portfolio

Corresponding author(s): Lu Liu, Zizhen Si

Last updated by author(s): Oct 10, 2025

Reporting Summary

Nature Portfolio wishes to improve the reproducibility of the work that we publish. This form provides structure for consistency and transparency in reporting. For further information on Nature Portfolio policies, see our Editorial Policies and the Editorial Policy Checklist.

Statistics

For all statistical analyses, confirm that the following items are present in the figure legend, table legend, main text, or Methods section.

n/a	Confirmed	
☐	☒	The exact sample size (*n*) for each experimental group/condition, given as a discrete number and unit of measurement
☐	☒	A statement on whether measurements were taken from distinct samples or whether the same sample was measured repeatedly
☐	☒	The statistical test(s) used AND whether they are one- or two-sided<br>*Only common tests should be described solely by name; describe more complex techniques in the Methods section.*
☐	☒	A description of all covariates tested
☐	☒	A description of any assumptions or corrections, such as tests of normality and adjustment for multiple comparisons
☐	☒	A full description of the statistical parameters including central tendency (e.g. means) or other basic estimates (e.g. regression coefficient) AND variation (e.g. standard deviation) or associated estimates of uncertainty (e.g. confidence intervals)
☐	☒	For null hypothesis testing, the test statistic (e.g. *F*, *t*, *r*) with confidence intervals, effect sizes, degrees of freedom and *P* value noted<br>*Give P values as exact values whenever suitable.*
☒	☐	For Bayesian analysis, information on the choice of priors and Markov chain Monte Carlo settings
☐	☒	For hierarchical and complex designs, identification of the appropriate level for tests and full reporting of outcomes
☐	☒	Estimates of effect sizes (e.g. Cohen's *d*, Pearson's *r*), indicating how they were calculated

Our web collection on statistics for biologists contains articles on many of the points above.

Software and code

Policy information about availability of computer code

Data collection	No software was used.
Data analysis	All data were analyzed using (version 20.0) (SPSS, USA).

For manuscripts utilizing custom algorithms or software that are central to the research but not yet described in published literature, software must be made available to editors and reviewers. We strongly encourage code deposition in a community repository (e.g. GitHub). See the Nature Portfolio guidelines for submitting code & software for further information.

Data

Policy information about availability of data

All manuscripts must include a data availability statement. This statement should provide the following information, where applicable:

- Accession codes, unique identifiers, or web links for publicly available datasets
- A description of any restrictions on data availability
- For clinical datasets or third party data, please ensure that the statement adheres to our policy

The data that support the findings of this study are available within the article and its supplementary materials. Additional raw data are available from the corresponding author upon reasonable request.

Research involving human participants, their data, or biological material

Policy information about studies with human participants or human data. See also policy information about sex, gender (identity/presentation), and sexual orientation and race, ethnicity and racism.

Reporting on sex and gender	n/a
Reporting on race, ethnicity, or other socially relevant groupings	n/a
Population characteristics	n/a
Recruitment	n/a
Ethics oversight	n/a

Note that full information on the approval of the study protocol must also be provided in the manuscript.

Field-specific reporting

Please select the one below that is the best fit for your research. If you are not sure, read the appropriate sections before making your selection.

☒ Life sciences ☐ Behavioural & social sciences ☐ Ecological, evolutionary & environmental sciences

For a reference copy of the document with all sections, see nature.com/documents/nr-reporting-summary-flat.pdf

Life sciences study design

All studies must disclose on these points even when the disclosure is negative.

Sample size	No statistical method was used to predetermine sample size. The sample sizes (n numbers) for each experiment were chosen based on common practices in the field and our extensive prior experience with the experimental models (e.g., mouse behavioral tests, isolated tissue preparations). Our sample sizes are similar to or exceed those reported in previously published studies investigating analogous biological questions, which ensures sufficient statistical power to detect significant effects. Each experiment was repeated independently at least three times to ensure the robustness of the findings.
Data exclusions	No data were excluded from the analyses reported in this study. All collected data are presented, and any technical failures or outliers that occurred during the experimental process were addressed by repeating the entire experiment, and thus were not included in the initial dataset for analysis.
Replication	All key experimental findings were successfully replicated. Specifically, each experiment was performed with a minimum of three independent biological replicates (e.g., using cells from different passages, tissues from different animals, or independently prepared primary neuronal cultures). Quantitative measurements were obtained from these independent replicates, and the results were consistent across all repetitions, confirming the reproducibility of our findings.
Randomization	Animals/cell culture plates were randomly allocated to experimental and control groups. However, due to the nature of some specific procedures (e.g., surgical interventions, genetic manipulations), complete randomization was not always feasible. In these cases, we ensured that animals or samples were matched for age, sex, and weight where applicable to control for potential covariates. For in vitro experiments, treatments were assigned systematically across plates to avoid positional biases.
Blinding	Blinding was not always possible during data collection due to the evident phenotypic changes induced by the treatments (e.g., obvious behavioral differences in animal groups). However, for outcome assessments that could be objectively quantified (e.g., analysis of Western blot band density, quantification of immunohistochemical images), the investigators were blinded to the group allocation during data analysis to prevent unconscious bias.

Reporting for specific materials, systems and methods

We require information from authors about some types of materials, experimental systems and methods used in many studies. Here, indicate whether each material, system or method listed is relevant to your study. If you are not sure if a list item applies to your research, read the appropriate section before selecting a response.

Materials & experimental systems

n/a	Involved in the study
☐	☒ Antibodies
☒	☐ Eukaryotic cell lines
☒	☐ Palaeontology and archaeology
☐	☒ Animals and other organisms
☒	☐ Clinical data
☒	☐ Dual use research of concern
☒	☐ Plants

Methods

n/a	Involved in the study
☒	☐ ChIP-seq
☒	☐ Flow cytometry
☒	☐ MRI-based neuroimaging

Antibodies

Antibodies used	The primary antibodies used in this study included LC3 (14600-1-AP, 1:1000), ADAR1 (14330-1-AP, 1:1000), and β-actin (66009-1-Ig, 1:5000) from Proteintech. GPX4 (ab125066, 1:1000), SIRT1 (ab110304, 1:1000) were purchased from Abcam. LAMP-2a (51-2200, 1:1000) from Invitrogen. p16INK4a (ZMS1072, 1:1000) was purchased from sigmaaldrich.
Validation	All primary antibodies used were commercially sourced. The specificity of each antibody was further confirmed in our experiments by the detection of a single band at the expected molecular weight in Western Blot analyses.

Animals and other research organisms

Policy information about studies involving animals; ARRIVE guidelines recommended for reporting animal research, and Sex and Gender in Research

Laboratory animals	This study involved laboratory animals. We used male C57BL/6J mice (purchased from Vital River Laboratories, China) and genetically modified mice on a C57BL/6 background. The Cdkn2aflox/flox (p16INK4a flox/flox) mice were (Cat. NO. NM-CKO-234392) were purchased from Shanghai Model Organisms Center, Inc. CaMKIIa-Cre mice were also provided by Shanghai model organisms. The Cdkn2aflox/flox (p16INK4a flox/flox) mice were mated with the CaMKIIa-driven Cre recombinase transgenic mice, and a neuronal-specific p16INK4a-deficient mouse model (p16INK4a-CKO mice) was generated. Atg7flox/flox mice (Cat. NO. NM-CKO-220047) were purchased from Shanghai Model Organisms Center, Inc. The ATG7flox/flox mice were bred with the CaMKIIa-driven Cre recombinase transgenic mice, and a neuronal-specific ATG7-deficient mouse model (ATG7-CKO mice) was generated.
Wild animals	This study did not involve wild animals.
Reporting on sex	Findings from this study apply to male mice only. Only male mice were used in all experiments to eliminate potential confounding effects of the estrous cycle on behavioral and biochemical outcomes. Sex was not assigned but was biologically determined. As data were collected from one sex only, sex-based analysis was not performed. The source data are not disaggregated by sex for this reason. The total number of animals used was 314 male mice.
Field-collected samples	This study did not involve samples collected from the field. All animals were laboratory-bred and housed in a specific pathogen-free (SPF) facility.
Ethics oversight	All animal experiments were performed in strict adherence to the National Institutes of Health Guidelines for the Care and Use of Laboratory Animals and received full ethical approval from the Institutional Animal Care and Use Committee at Ningbo University (Approval ID: NBU-IACUC-2021-10476).

Note that full information on the approval of the study protocol must also be provided in the manuscript.

Plants

Seed stocks	n/a
Novel plant genotypes	n/a
Authentication	n/a
